# Global Epidemiology of Mental Disorders: What Are We Missing?

**DOI:** 10.1371/journal.pone.0065514

**Published:** 2013-06-24

**Authors:** Amanda J. Baxter, George Patton, Kate M. Scott, Louisa Degenhardt, Harvey A. Whiteford

**Affiliations:** 1 School of Population Health, University of Queensland, Herston, Australia; 2 Queensland Centre for Mental Health Research, Wacol, Australia; 3 Department of Paediatrics, University of Melbourne, Melbourne, Australia; 4 Murdoch Childrens Research Institute, Melbourne, Australia; 5 Department of Psychological Medicine, University of Otago, Dunedin, New Zealand; 6 National Drug and Alcohol Research Centre, University of New South Wales, Sydney, Australia; 7 Centre for Health Policy, Programs and Economics, School of Population Health, University of Melbourne, Melbourne, Australia; Aga Khan University, Pakistan

## Abstract

**Background:**

Population-based studies provide the understanding of health-need required for effective public health policy and service-planning. Mental disorders are an important but, until recently, neglected agenda in global health. This paper reviews the coverage and limitations in global epidemiological data for mental disorders and suggests strategies to strengthen the data.

**Methods:**

Systematic reviews were conducted for population-based epidemiological studies in mental disorders to inform new estimates for the global burden of disease study. Estimates of population coverage were calculated, adjusted for study parameters (age, gender and sampling frames) to quantify regional coverage.

**Results:**

Of the 77,000 data sources identified, fewer than 1% could be used for deriving national estimates of prevalence, incidence, remission, and mortality in mental disorders. The two major limitations were (1) highly variable regional coverage, and (2) important methodological issues that prevented synthesis across studies, including the use of varying case definitions, the selection of samples not allowing generalization, lack of standardized indicators, and incomplete reporting. North America and Australasia had the most complete prevalence data for mental disorders while coverage was highly variable across Europe, Latin America, and Asia Pacific, and poor in other regions of Asia and Africa. Nationally-representative data for incidence, remission, and mortality were sparse across most of the world.

**Discussion:**

Recent calls to action for global mental health were predicated on the high prevalence and disability of mental disorders. However, the global picture of disorders is inadequate for planning. Global data coverage is not commensurate with other important health problems, and for most of the world's population, mental disorders are invisible and remain a low priority.

## Introduction

The Global Burden of Disease (GBD) study published in 1996 showed that neuro-psychiatric disorders account for more than a quarter of all health loss due to disability, more than eight times greater than that attributed to coronary heart disease and 20-fold greater than cancer [1]. These findings highlighted for the first time the central place of mental disorders in population health as well as a need for a response from health service systems. Sound epidemiological information around mental disorders is an essential starting point for that policy response.

The pursuit of a comprehensive picture of mental disorders, however, has varied enormously across regions and across disorders. Global surveillance systems such as the WHO Stepwise Approach to Chronic Disease Factor Surveillance (STEPS) [2], the Multiple Indicator Cluster Surveys (MICS) [3] and the MEASURE Demographic and Health Surveys (DHS) Project [4] now cover a number of major causes of disease burden in low income countries. These systems provide little coverage of mental disorders and there is no comparable global data collection system in place for mental disorders. Moreover there is as yet no global standard for collection of health measures or repository for cross-national data on mental disorders. Lack of standardized indicators for this large group of disorders hinders the development of a comprehensive global health agenda [5].

In 2007 a new Global Burden of Disease Study (GBD 2010) commenced. Funded by the Bill and Melinda Gates Foundation, its aim was to make comprehensive burden estimates for over 290 disease and injury categories by age and gender in 21 world regions (see Figure S1 for GBD 2010 world region classifications). An important evolution of this latest GBD study is that new estimates for disease burden would be calculated within a framework driven by the best available epidemiological data [6]. A series of systematic reviews was therefore conducted to identify epidemiological studies describing the prevalence, incidence and course of illness for mental disorders that underpin the new burden estimates. This study provides an opportunity to consider the adequacy of current data to guide a global mental health agenda.

Detailed reports of the process used to conduct our systematic reviews have been published elsewhere [7], [8], [9]. The aim of this paper is to report an overview of the strengths and limitations found in the current epidemiological research on mental disorders, and from this appraisal arrive at strategies for strengthening the data needed to inform planning and public health policy.

Overall, our review series encompassed seven classes of disorders: depressive disorders; anxiety disorders; schizophrenia; bipolar disorder; eating disorders; childhood behavioural disorders (CBD) and autistic spectrum disorders (ASD). In considering adequacy of epidemiological measures for mental disorders, we found the challenges associated with collating data differed for childhood disorders compared to other mental disorders. The issues of compiling epidemiological data on mental disorders for children deserves greater attention than is possible within this paper and here we focus on our findings in relation to the most common disorders in adult populations.

## Materials and Methods

### Defining disorders

Our review included depressive disorders (major depression and dysthymia), anxiety disorders (‘any’ anxiety disorder), bipolar disorder, schizophrenia and eating disorders (anorexia nervosa and bulimia) defined as meeting clinical diagnostic threshold (see Table S2 for more detail). Data were sought for specific disorders (e.g. major depression and dysthymia, anorexia and bulimia nervosa) with the exception of anxiety disorders which, due to their high co-morbidity, were defined as meeting criteria for ‘any’ anxiety disorder.

To compare data availability, we grouped the mental disorders into broad ‘prevalence’ categories as the frequency of a disorder in the population is relevant to the methodological approach taken to identify cases and capture information on disease prevalence and incidence. Depressive disorders and anxiety disorders were considered *high prevalence disorders* for the purpose of this report while bipolar, schizophrenia and eating disorders were classified as *low prevalence disorders*.

### Systematic review

Measures of prevalence, incidence, remission, and excess all-cause mortality are required to derive prevalent and incident disability for guiding health service delivery and intervention strategies (see Appendix S1 for descriptions of epidemiologic measures). We conducted a series of systematic reviews to identify these data based on an iterative strategy as recommended by the Meta-analysis of Observational Studies in Epidemiology (MOOSE) group [10]. Electronic databases (Medline, Embase and PsychoINFO) were interrogated using broad search strings developed with the assistance of research librarians. Secondary searches of non-indexed journals and alternative academic databases were conducted for region-specific data, these methods are described in more detail in Appendix S2. Reference lists for review articles, editorials and resource books were manually scrutinized and online searches conducted for data such as government surveys, international collaborative research projects, and research theses. Throughout this process the shortlisted studies were critically reviewed through expert consultation. Details on the prevalence systematic reviews for bipolar disorder [7], major depressive disorder [8], anxiety disorders [9] and eating disorders [11] have been accepted or published in peer-reviewed journals.

There were minor differences in the review methodology for schizophrenia, which was completed prior to the GDB2010 study, including use of broader inclusion criteria. McGrath, Saha and colleagues [12], [13], [14] conducted reviews for the epidemiology of schizophrenia which formed the starting point for data collection in GDB2010. Further detail on methods and data sources are reported in Appendix S2 and Table S2.

### Inclusion/exclusion criteria

Since GBD estimates were made at national and regional level, data were required that described disease epidemiology in the broader population. Due to the scarcity of community-based studies for less common disorders (schizophrenia, bipolar and eating disorders) we included remission and mortality studies based on clinical samples with naturalistic follow-up. Studies were sought that a) defined mental disorders according to internationally accepted diagnostic criteria, i.e. the Diagnostic and Statistical Manual of Mental Disorders (DSM) [15] or International Classification of Mental and Behavioural Disorders (ICD) [16], and b) were homogenously categorized. Only data reported by primary sources were included, and sufficient detail on study method and findings was required to assess whether the above criteria were met. Studies published between 1980 and 2008 were included, with earlier and later reports added if provided through expert consultation. No limitation was set on language of publication or sample size (see Appendix S2 for more information on study inclusion criteria).

### Calculating population coverage

To give a sense of how complete the available data were, in terms of GBD world regions, we calculated the proportion of regional populations for which prevalence data were reported and adjusted estimates for age, sex, and sampling frames of the studies that provided data. Calculations were based on the population aged between 18 and 80 years to allow comparison between the disorder groups. To illustrate, if one study was found for Eastern Sub-Saharan Africa, capturing men and women from Ethiopia aged 60 to 80 years, the coverage was considered representative of the corresponding proportion of that country's population aged 60–80 years, relative to the population of the region. Where the study sampling frame was sub-national, for example one major city, coverage was adjusted by the population of that community relative to, first the country, then the region. These estimates are referred to as the population coverage of available data for specific disorders. Average coverage refers to the mean of disorder-specific regional coverage, for example data coverage for high prevalence disorders is the simple mean of the coverage estimates for anxiety, MDD and dysthymia. All estimates were calculated with Microsoft Excel and based on country population data from the United Nations (UN) Population Division for 2005. These calculations are described in more detail in Appendix S3, including specific examples.

To compare the availability of data by country economic status, we calculated the simple proportion of the high income (HI) countries and the low- to middle-income (LMI) countries that provided any prevalence data. Income categories are based on the World Bank's classification for gross national income (GNI) per capita (http://data.worldbank.org/about/country-classifications/country-and-lending-groups).

## Results

Our initial search identified almost 77,000 epidemiological studies related to higher and low prevalence mental disorders (see table 1). As expected, the majority of the initial studies focused on prevalence of mental disorders and related issues in HI countries, particularly in Western Europe and North America. While research into population mental disorders is increasing in countries in Asia and Latin America, studies remained scarce for much of Africa and Central and Eastern Europe.

**Table 1:** Results of systematic reviews conducted to identify community-representative epidemiological data for higher and low prevalence mental disorders.

Mental disorders	Number of electronic databases data sources	Number of data sources, other	Data sources used in deriving burden estimates*			
			Prevalence	Incidence	Remission	Mortality
**High Prevalence Disorders**						
Depressive disorders	35,579	36	267	6	6	10
Anxiety disorders	22,423	34	96	3	5	2
**Low Prevalence Disorders**						
Bipolar disorder	2,442	44	32	2	0	5
Schizophrenia	3,673	14	51	33	12	32
Eating disorders	12,777	4	33	7	21	11
**Total**	76,894**	132	479	51	44	60

* Number of data sources by disorder. Note that some studies report data for more than one disorder.

** In total 96,349 data sources were identified for the review series (ie. high and low prevalence disorders and disorders with onset in childhood).

With data on mental disorders already limited in many world regions, the vast number of the identified studies that did not meet inclusion criteria further reduced the scope of useable data. Table 1 shows that less than 1% of the studies identified were suitable for use in developing a global epidemiological picture for mental disorders. Four main limitations led to exclusion of these studies: 1) inability to fulfill currently accepted standardized definitions for mental disorders; 2) non-representative samples making the generalization of findings not possible; 3) use of measures unable to provide comparable estimates between studies and 4) incomplete reporting of study methods and results. The remaining studies also varied in terms of methods and completeness of reporting, and whilst these 1% of studies represent the best quality data available, we found similar limitations to some degree across even these higher quality studies

### Global coverage of prevalence data

The population coverage of prevalence data, in terms of data completeness by sex, age and national-representativeness, are shown in table 2. Regional and world estimates for population coverage reflect the proportion of each population for which the available data is considered representative. Coverage is reported for specific disorders with the exception of anorexia and bulimia for which the data were very similar, hence the data coverage for these disorders are aggregated under ‘eating disorders’.

**Table 2:** Estimated global coverage* of prevalence data for mental disorders by Global Burden of Disease 2010 Study world region.

GBD World Region**	Regional population in 2005 (,000)	High prevalence disorders			Low prevalence disorders		
	(18–80 yrs)	Major depression	Dysthymia	Anxiety disorders	Schizophrenia	Bipolar disorder	Eating disorders
Asia Pacific, High Income	140,611	80.8%	1.0%	93.1%	71.6%	3.8%	23.1%
Asia, Central	48,459	0.0%	0.0%	0.0%	0.0%	0.0%	0.0%
Asia, East	968,141	12.2%	8.3%	2.5%	15.6%	8.4%	7.9%
Asia, South	887,704	1.7%	0.0%	4.9%	6.3%	0.0%	0.0%
Asia, Southeast	368,908	14.5%	0.0%	15.5%	0.4%	0.0%	0.0%
Australasia	17,792	100.0%	100.0%	100.0%	85.1%	100.0%	16.4%
Caribbean	26,964	9.1%	0.0%	0.0%	28.3%	0.0%	0.0%
Europe, Central	91,890	16.0%	0.0%	25.5%	0.0%	0.0%	18.8%
Europe, Eastern	164,965	23.6%	22.9%	22.3%	1.3%	1.7%	0.0%
Europe, Western	310,486	73.6%	7.5%	81.8%	12.6%	19.0%	57.3%
Latin America, Andean	29,663	0.0%	0.0%	0.0%	0.0%	0.0%	0.0%
Latin America, Central	131,753	49.8%	46.0%	69.7%	0.7%	34.5%	71.0%
Latin America, Southern	39,110	16.5%	0.0%	28.4%	0.0%	11.6%	0.0%
Latin America, Tropical	125,791	9.7%	6.4%	6.4%	0.0%	6.4%	21.1%
North Africa/Middle East	249,810	47.0%	23.1%	43.7%	0.0%	14.5%	0.0%
North America, High Income	239,174	100.0%	90.2%	93.4%	89.8%	89.8%	89.8%
Oceania	4,725	0.0%	0.0%	0.0%	0.4%	0.0%	0.0%
Sub-Saharan Africa, Central	39,586	0.0%	0.0%	0.0%	0.0%	0.0%	0.0%
Sub-Saharan Africa, East	153,193	1.3%	0.7%	0.4%	6.4%	0.9%	0.1%
Sub-Saharan Africa, Southern	39,988	0.1%	0.1%	73.6%	<0.1%	0.0%	0.0%
Sub-Saharan Africa, West	147,840	46.6%	46.6%	46.6%	0.0%	47.0%	0.0%
World	4,227,400	35.4%	29.4%	44.2%	14.2%	12.9%	15.2%

* Coverage: % of population represented by prevalence studies for mental disorders, adjusted for study age-ranges, gender-coverage and sub-national sampling frames.

** GBD World Region: see Figure S1 for more information on world regions used in the Global Burden of Disease Study 2010.

#### High prevalence disorders

**Figure 1:**
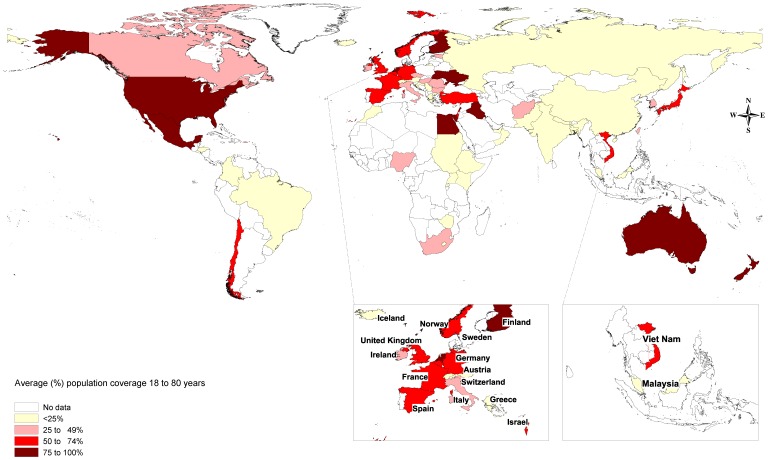
Population coverage of prevalence data for common mental disorders: averaged across major depressive disorder, dysthymia and anxiety disorders.

The most complete data were from North America and Australasia with each of the three common conditions having greater than 75% coverage in adults between 18 and 80 years of age. There was moderate to good coverage of Western Europe and the high income countries of the Asia Pacific for both major depression (MDD) and anxiety but not for dysthymia. In other regions coverage of common mental disorders was poor to absent. For 64% of the world's population, aged between 18 and 80 years, there was no information on prevalence for common mental disorders (see table 2). Figure 1 shows the data coverage for high prevalence disorders, averaged across MDD, dysthymia and anxiety disorders and adjusted for national-representativeness of study samples (i.e. age-groups, sex and sampling frames).

Studies for MDD were available from 53 countries, comprising almost one half of all HI countries and one sixth of all LMI countries. Dysthymia was reported in 27 countries. One quarter of all HI countries and one in 14 of all LMI countries provided at least some prevalence data. Forty-five countries provided data for ‘any’ anxiety disorders, including one quarter of all HI countries and one sixth of all LMI countries.

#### Low prevalence disorders

**Figure 2:**
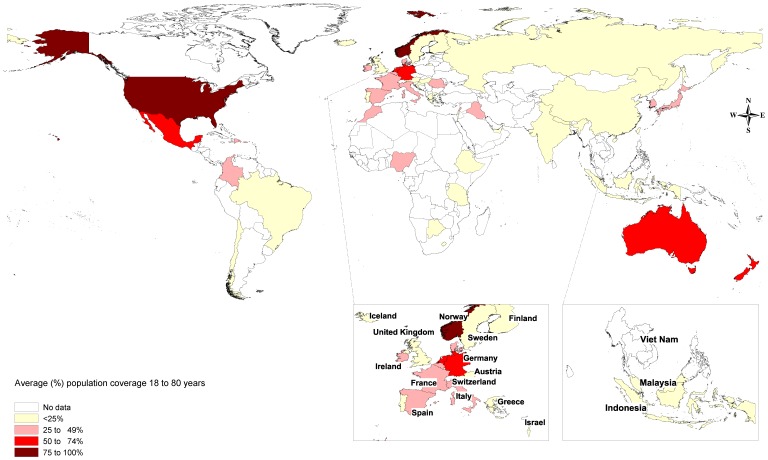
Average population coverage of prevalence data for low prevalence disorders: averaged across schizophrenia, bipolar disorder and eating disorders.

North America provided the most complete data for low prevalence disorders with each of schizophrenia, bipolar and eating disorders having about 90% coverage in adults aged 18 to 80 years. There was good coverage for schizophrenia and bipolar disorder in Australasia but not for eating disorders (see table 2). Data for low prevalence disorders in other regions was scarce with data missing for almost 86% of the global population. Figure 2 shows the average population coverage for low prevalence disorders based on the simple mean of coverage in adults for bipolar, schizophrenia and eating disorders. The regional population coverage for specific disorders is shown in table 2.

The global data for schizophrenia were more widely distributed (across 27 countries) compared with the global data for bipolar and eating disorders (24 and 22 countries, respectively). This can be attributed, at least in part, to the broader inclusion rules that allowed schizophrenia studies based on clinical samples. One quarter of all HI countries and one in fourteen LMI countries provided data for schizophrenia. One fifth of HI countries provided data for bipolar disorders in comparison with one in fifteen of the LMI countries. For eating disorders, data were found for one in four of all HI countries but only one in 26 of all LMI countries.

### Global coverage of incidence data

High income countries in North America and Western Europe provided the majority of the incidence studies for common mental disorders. Incidence data for MDD and dysthymia were almost entirely limited to the United States and Canada, with the exception of one Ethiopian study for major depression in a rural sample [17]. Incidence for ‘any’ anxiety disorder was available from only three countries, specifically the Netherlands, Norway and United Arab Emirates.

Of the low prevalence disorders more incidence studies were found for schizophrenia compared with bipolar or eating disorders although the schizophrenia studies identified in a previous review [12], primarily relied on clinical samples which were less likely to be included for other disorders due to more stringent inclusion rules. Incidence rates for schizophrenia were found for 13 countries (12 HIC and 1 LMIC). One country, the USA, reported incidence data for bipolar disorders. Six countries provided incidence data for eating disorders, all of which were classified as HI and all, with the exception of one study from the USA [18], limited to females only.

### Global coverage of remission data

The most complete remission data for high prevalence disorders remission was from HI countries in North America and Western Europe. The single study outside these regions that reported remission was from a LMI country, examining anxiety in children and adolescents from India [19].

Seventeen countries (11 HIC and 6 LMIC) provided remission data for schizophrenia, of which one half were regional studies comprising the International Study of Schizophrenia (ISoS) [20]. All data for eating disorders were obtained from HI countries, and of the seven countries represented, all but one study [21] were conducted in Western Europe, North America or Australasia. More than one half of studies reported remission in females only. A dearth of data was found for remission in community-cases of bipolar disorder.

### Global coverage of mortality data

Seven countries reported mortality in major depression: the USA, UK, Finland, the Netherlands, Sweden, Australia and somewhat unexpectedly, Ethiopia, representing just over one in ten of the HI countries and less than one percent of all LMI countries. Community-representative mortality data were available from only two HIC (USA and Finland) for anxiety disorders and dysthymia.

Mortality data were found for schizophrenia in 21 countries (16 HIC and 5 LMIC). In contrast, mortality data for bipolar and eating disorders was only available from HI countries with a dearth of information available from populations in LMI countries. Five HI countries in Asia Pacific, Western Europe and North America reported mortality in bipolar disorder. Anorexia nervosa was the only eating disorder for which we found mortality data, with data from seven countries across Western Europe, North America and Asia East.

## Discussion

Overall, our review found that four of the 21 GBD world regions lacked any data on mental disorders: Central Asia, Central Sub-Saharan Africa, Andean Latin America and Oceania. Nationally comparable information on mental disorders in LMI countries was notable by its absence. Australasia and North America provided the most complete prevalence data for common mental disorders. Globally, the population coverage for dysthymia (29%) was noticeably lower than that for MDD and anxiety disorders (35% and 44%, respectively). The majority of information on incidence, remission and mortality was found in Western Europe and North America while data were scarce to nonexistent for other regions, and particularly for LMI countries.

Coverage for the low prevalence disorders was similar across disorders, but represented less than a sixth of the global population. North America had the most complete information on prevalence of less common mental disorders. Whilst Australasia had reasonably complete data coverage for schizophrenia and bipolar, nationally-comparable information on prevalence of eating disorders was poor. Paucity of population-representative studies on incidence, remission and mortality in low prevalence disorders meant that we had to broaden the review to include studies with clinical samples, albeit those with naturalistic follow-up. Data for incidence and outcome in low prevalence disorders was largely from HIC in North America and Western Europe, with little information available from LMIC.

This overview illustrates both the global and regional limitations in data coverage. These limitations are due in part to the absence of studies, particularly in poorly resourced countries. In addition to low prioritization of research in mental disorders, however, there was wide variation in study methods which impact on the accuracy of epidemiological measures. Consistency in case definition and measurement, and appropriate sampling and reporting procedures, were central methodological barriers in collating information on mental disorders.

The reviews sought epidemiological data for mental disorders defined according to DSM or ICD diagnostic criteria. As with many medical disorders, the symptoms and severity of mental disorders present along a continuous spectrum rather than as a dichotomous paradigm [22]. However health-care planning is predicated on clinically-relevant cases and therefore epidemiological measures used in health-care planning must reflect cases that meet clinical threshold. The instruments used may vary from brief symptom checklists to fully-standardized surveys, and finally, the gold standard of clinical interview [23]. Symptom checklists are inexpensive to administer and demonstrate good sensitivity but generally have low specificity and poor positive predictive power for clinical caseness [24], [25], [26], [27], so when used in the absence of diagnostic instruments, these can lead to elevated estimates for prevalence [8]. Global coverage of useable prevalence data was substantially reduced after excluding studies that used only symptom checklists [28], [29], [30], [31], [32], resulting in loss of prevalence data for a number of countries, including Ghana, the Philippines and Poland

Beyond prevalence, consistent and explicit disorder definitions are essential to understanding disease course as specific disorders within a diagnostic category have different risk for health outcomes. For example, depression is associated with a 50% greater risk of premature mortality when cases are defined using a imprecise definition of ‘depressive disorder’ [33]. Yet, when studies used explicit definitions of major depression (MDD) and dysthymia, a clear difference was found between MDD, which was associated with a 92% greater risk of mortality, and dysthymia which had no association with excess mortality [33].

National mental health surveys provide an ideal framework for collecting epidemiological data but require substantial investment and a large research team. Regional studies within countries may provide estimates generalizable to the broader population but not from purposive sampling frames linked, for example, groups to conflict, with elevated prevalence estimates for anxiety disorders and depression [34], [35].

National surveys often rely on household sampling, but extending case-finding to non-household settings (e.g. hospitals and prisons) and to marginalized groups (e.g. homeless populations) and accessing additional sources such as welfare records will lead to more comprehensive data. People with mental health or drug use problems are probably less likely to be available for interview, or to agree to an interview if contacted. Marginalized groups (e.g. homeless people and prisoners) often have high rates of mental disorders and drug use and their non-inclusion in household population surveys can affect the overall estimates for low prevalence disorders [36]. The expense of incorporating these populations is a particular challenge in low income countries.

The experience of general population surveys in Nordic countries has shown the value of data linkage studies. Bias due to non-response and attrition can be identified, as illustrated by the HUSK study finding that one third of persons receiving disability benefits for mental illness refused participation in a cohort study of adults in western Norway [37]. Information on non-participants can inform imputation of missing data to arrive at more accurate epidemiological estimates. Moreover, linked records can provide more accurate information on individuals' treatment (or lack thereof) which is relevant for informing policy as there is poor alignment between self-reported treatment and use of pharmacotherapies [38].

Standard indicators of mental disorder have yet to be defined. To illustrate, we found prevalence measures for current (point/30-day), 3-month, 6-month, past-year and lifetime prevalence. While observational cross-sectional studies are ideal for measuring current prevalence, a prolonged period of recall for establishing lifetime prevalence results in low estimates for prevalence [39], [40] as querying of past symptoms can elicit insufficient detail of onset and co-occurrence of symptoms and impairment to establish clinical threshold [41]. This lack of standardization highlights an urgent need for engagement between stakeholders including WHO, other relevant United Nations agencies and the World Psychiatric Association in setting internationally comparable indicators for mental disorders.

Incidence, remission and duration of mental disorders are most accurately gauged with data from prospective cohort studies [23]. Birth cohort studies from New Zealand [42], [43] and Finland [44] have provided incidence and remission data for common mental disorders. Similar data was reported from longitudinal follow-up of community surveys in Canada [45], [46], the USA [47], [48], and the Netherlands [49], [50], [51]. Childhood surveys with a longitudinal component were available from the United Kingdom, Canada [52] and Sweden [53]. With the limited number of available prospective studies [54], inconsistency between measures is a major issue in trying to compare and pool estimates for remission and duration [55].

Two further limitations in the current literature were quality of reporting and dissemination of findings, and this can have implications for inferences drawn from study findings. While reporting in the peer-reviewed literature has improved over the past two decades, it is surprising that a number of studies still do not clearly define their outcomes of interest, period of data collection, target population or estimates of uncertainty. These omissions are easily rectified and, when study results are clearly communicated, are more likely to be taken up as a reliable information source. A second issue was lack of published data from LMI countries where studies are not only hindered by lack of funding and research support, but also difficulty in publishing in peer-reviewed journals. Language difficulties hamper publication outside the region, and local journals are frequently non-indexed. Of 222 indexed psychiatric journals, fewer than 5% originated from middle-income countries and none from low-income countries, despite the publication of more than 118 psychiatric journals in LMIC countries [56]. The World Psychiatric Association (WPA) is attempting to alleviate this bottleneck through developing regional editorial capacity in LMI countries to meet requirements for full indexation [57].

A coordinated approach is needed to improve the quality and quantity of data produced, bringing epidemiological research in the field of mental disorders into line with other fields of medicine. Psychiatric research in many LMI countries is considered a low priority in the face of overwhelming competition for resources. Somewhat heroic assumptions about LMI epidemiology are therefore made based on data from other populations. Yet populations are characterized by different cultural, environmental and genetic factors. Until further research is conducted and disseminated we will not know the true extent of mental disorders and their outcome in these groups. It may be that burden of mental disorders is currently under-represented in LMIC, a serious issue for population measures of mental disorders and the policymakers who use them, given LMIC populations account for more than 80% of the world's populace. One opportunity for expanding global coverage in these populations may be the nesting of mental health data collection within general health surveys already operating in LMIC such as the DHS project and the Stepwise Approach to Chronic Disease Factor Surveillance [5].

The WMHS Collaboration has also started to address lack of research in LMI countries through assisting researchers to access funding and technical assistance to conduct population surveys. National mental health surveys have now been conducted in over 28 countries with the aim of informing public health policy [58], [59]. In addition to providing data for under-represented populations, such large-scale mental health surveys offer a valuable but currently under-utilised opportunity to collect longitudinal data through follow-up of sub-samples identified. Prospectively collected data are requisite to characterizing the natural course of illness, including incidence, remission and duration, all relevant for estimating the resources needed to provide interventions. Beyond data on disease trajectory, prospective studies also provide information on duration of untreated illness. Increased time to treatment is associated with poorer outcomes hence understanding the lag between onset of symptoms and treatment-seeking is necessary to reducing duration of illness.

Collection and collation of cross-national information on mental disorders does not currently fall under a central aegis resulting in non-standardized research methodology and inequitable population coverage. WHO and other UN agencies have developed standardized surveillance systems [2], [3] which produce a comparable set of indicators for a number of diseases. A similar systematic approach to measurement and data collection is needed for mental disorders. There would be potential for collating and disseminating epidemiological research through a central online site. A good example of what could be achieved is that developed by the International Agency for Research on Cancer under the auspice of WHO (see http://www-dep.iarc.fr/). Such a repository has commenced for schizophrenia research (http://www.intersect.org.au/australian-schizophrenia-research-bank) but nothing yet exists across the range of mental disorders.

## Conclusion

The call to action for global mental health [60], [61] was predicated on the evidence for high prevalence and disability in mental disorders and inequity of service provision. However, our understanding of the distribution and outcome of mental disorders is inadequate for effective policy- and service-planning. Although some efforts are being made to strengthen epidemiological research in the international mental health community [57], [58], [59] good data are absent for most of the world's population. The result is that for most parts of the globe mental disorders will remain invisible and a low priority compared to other major global health agendas.

## Supporting Information

Figure S1 **GBD2010 world region classifications.**
(DOCX)

Table S1 Mental disorder classes included in GBD2010.
(DOCX)

Table S2 References for studies that provided data included in coverage calculations.
(DOCX)

Appendix S1 **Epidemiological measures required as data input.**
(DOCX)

Appendix S2 **Systematic search protocol.**
(DOCX)

Appendix S3 **Calculating population coverage.**
(DOCX)
