# Supplementary material for: Global Epidemiology of Mental Disorders: What Are We Missing?
Source: PLoS One. 2013 Jun 24;8(6):e65514. doi: 10.1371/journal.pone.0065514 (PMC3691161; doi:10.1371/journal.pone.0065514)
Supplement: Figure S1 — GBD2010 world region classifications. (DOCX) [file pone.0065514.s001.docx]

# Figure S1: GBD2010 world region classifications

*Source of regional information: GBD Study Operations Manual, January 20 2009. Prepared by Investigators from Harvard University; Institute for Health Metrics and Evaluation at the University of Washington; Johns Hopkins University; University of Queensland; World Health Organization; Available at: http://www.globalburden.org/GBD_Study_Operations_Manual_Jan_20_2009.pdf.*
