# Supplementary material for: Global Epidemiology of Mental Disorders: What Are We Missing?
Source: PLoS One. 2013 Jun 24;8(6):e65514. doi: 10.1371/journal.pone.0065514 (PMC3691161; doi:10.1371/journal.pone.0065514)
Supplement: Table S1 — Mental disorder classes included in GBD2010. (DOCX) [file pone.0065514.s002.docx]

# Table S1: Mental disorder classes included in GBD2010.

Seven major categories of mental disorders were captured in the Global Burden of Disease Study 2010 (GBD 2010). In these analyses we consider the high prevalence and low prevalence disorders. Childhood disorders* will be discussed separately.

| Mental disorder categories | Specific disorders |
| --- | --- |
| High prevalence disorders |  |
| 1. Depressive disorders | Major depression and depressive disorder-NOS  Dysthymic disorder |
| 2. Anxiety disorders | Any anxiety disorder, including generalized anxiety disorder (GAD), panic disorder, agoraphobia, social phobia, specific phobias, obsessive-compulsive disorder (OCD), posttraumatic stress disorder (PTSD), separation anxiety disorder (SAD) and anxiety disorders-NOS |
| Low prevalence disorders |  |
| 3. Schizophrenia | Schizophrenia |
| 4. Bipolar disorders | Bipolar disorders, including bipolar I, bipolar II, cyclothymic disorder and bipolar disorders -NOS |
| 5. Eating disorders | Anorexia nervosa  Bulimia nervosa and eating disorders-NOS) |
| Disorders with onset in childhood* |  |
| 6. Childhood behavioural disorders | Attention-deficit/hyperactivity disorder  Conduct disorder, oppositional-defiant disorder and disruptive behaviour disorder-NOS |
| 7. Pervasive developmental disorders | Autistic disorder  Asperger's disorder and pervasive developmental disorders-NOS |
