# Supplementary material for: Global Epidemiology of Mental Disorders: What Are We Missing?
Source: PLoS One. 2013 Jun 24;8(6):e65514. doi: 10.1371/journal.pone.0065514 (PMC3691161; doi:10.1371/journal.pone.0065514)
Supplement: Table S2 — References for studies that provided data included in coverage calculations. (DOCX) [file pone.0065514.s003.docx]

**Table S2: References for studies that provided data included in coverage calculations**

**Number of references may not equal number of studies reported in text as multiple study results (eg cross-national consortiums such as the WHMS) are covered by a single reference.*

*** WMHS data were obtained for overall anxiety disorders from the WMHS consortium while data for MDD and dysthymia were collated from the peer-reviewed literature.*

1. High prevalence disorders

| **Region** | **References for prevalence data sources** |
| --- | --- |
| **Major Depression** | |
| Asia Pacific, High Income | [[1](#_ENREF_1),[2](#_ENREF_2),[3](#_ENREF_3),[4](#_ENREF_4),[5](#_ENREF_5),[6](#_ENREF_6),[7](#_ENREF_7)] |
| Asia, East | [[7](#_ENREF_7),[8](#_ENREF_8),[9](#_ENREF_9),[10](#_ENREF_10),[11](#_ENREF_11),[12](#_ENREF_12),[13](#_ENREF_13)] |
| Asia, South | [[13](#_ENREF_13),[14](#_ENREF_14),[15](#_ENREF_15)] |
| Asia, Southeast | [[16](#_ENREF_16)] |
| Australasia | [[17](#_ENREF_17),[18](#_ENREF_18),[19](#_ENREF_19),[20](#_ENREF_20),[21](#_ENREF_21)] |
| Caribbean | [[22](#_ENREF_22),[23](#_ENREF_23)] |
| Europe, Central | [[24](#_ENREF_24),[25](#_ENREF_25),[26](#_ENREF_26)] |
| Europe, Eastern | [[27](#_ENREF_27),[28](#_ENREF_28),[29](#_ENREF_29)] |
| Europe, Western | [[13](#_ENREF_13),[24](#_ENREF_24),[30](#_ENREF_30),[31](#_ENREF_31),[32](#_ENREF_32),[33](#_ENREF_33),[34](#_ENREF_34),[35](#_ENREF_35),[36](#_ENREF_36),[37](#_ENREF_37),[38](#_ENREF_38),[39](#_ENREF_39),[40](#_ENREF_40),[41](#_ENREF_41),[42](#_ENREF_42),[43](#_ENREF_43),[44](#_ENREF_44),[45](#_ENREF_45),[46](#_ENREF_46),[47](#_ENREF_47),[48](#_ENREF_48),[49](#_ENREF_49),[50](#_ENREF_50),[51](#_ENREF_51),[52](#_ENREF_52),[53](#_ENREF_53),[54](#_ENREF_54),[55](#_ENREF_55),[56](#_ENREF_56)] |
| Latin America, Central | [[24](#_ENREF_24),[57](#_ENREF_57),[58](#_ENREF_58)] |
| Latin America, Southern | [[13](#_ENREF_13),[24](#_ENREF_24),[59](#_ENREF_59)] |
| Latin America, Tropical | [[13](#_ENREF_13),[38](#_ENREF_38),[60](#_ENREF_60),[61](#_ENREF_61)] |
| North Africa/Middle East | [[13](#_ENREF_13),[24](#_ENREF_24),[62](#_ENREF_62),[63](#_ENREF_63),[64](#_ENREF_64),[65](#_ENREF_65),[66](#_ENREF_66),[67](#_ENREF_67),[68](#_ENREF_68)] |
| North America, High Income | [[13](#_ENREF_13),[24](#_ENREF_24),[69](#_ENREF_69),[70](#_ENREF_70),[71](#_ENREF_71),[72](#_ENREF_72),[73](#_ENREF_73),[74](#_ENREF_74),[75](#_ENREF_75),[76](#_ENREF_76),[77](#_ENREF_77),[78](#_ENREF_78),[79](#_ENREF_79),[80](#_ENREF_80),[81](#_ENREF_81),[82](#_ENREF_82),[83](#_ENREF_83)] |
| Sub-Saharan Africa, East | [[84](#_ENREF_84),[85](#_ENREF_85),[86](#_ENREF_86),[87](#_ENREF_87),[88](#_ENREF_88),[89](#_ENREF_89)] |
| Sub-Saharan Africa, Southern | [[90](#_ENREF_90),[91](#_ENREF_91)] |
| Sub-Saharan Africa, West | [[13](#_ENREF_13),[92](#_ENREF_92),[93](#_ENREF_93),[94](#_ENREF_94),[95](#_ENREF_95),[96](#_ENREF_96)] |
| **Dysthymic disorder** | |
| Asia Pacific, High Income | [[1](#_ENREF_1),[2](#_ENREF_2),[3](#_ENREF_3),[4](#_ENREF_4),[5](#_ENREF_5)] |
| Asia, East | [[7](#_ENREF_7),[10](#_ENREF_10),[12](#_ENREF_12)] |
| Australasia | [[17](#_ENREF_17),[18](#_ENREF_18),[20](#_ENREF_20),[97](#_ENREF_97)] |
| Europe, Eastern | [[28](#_ENREF_28),[29](#_ENREF_29)] |
| Europe, Western | [[30](#_ENREF_30),[32](#_ENREF_32),[33](#_ENREF_33),[37](#_ENREF_37),[39](#_ENREF_39),[45](#_ENREF_45),[49](#_ENREF_49),[55](#_ENREF_55)] |
| Latin America, Central | [[98](#_ENREF_98)] |
| Latin America, Tropical | [[60](#_ENREF_60),[61](#_ENREF_61)] |
| North Africa/Middle East | [[63](#_ENREF_63),[65](#_ENREF_65),[66](#_ENREF_66)] |
| North America, High Income | [[69](#_ENREF_69),[70](#_ENREF_70),[74](#_ENREF_74),[77](#_ENREF_77),[83](#_ENREF_83),[99](#_ENREF_99)] |
| Sub-Saharan Africa, East | [[86](#_ENREF_86)] |
| Sub-Saharan Africa, Southern | [[90](#_ENREF_90)] |
| Sub-Saharan Africa, West | [[95](#_ENREF_95)] |
| **Anxiety disorders** | |
| Asia Pacific, High Income | [[1](#_ENREF_1),[100](#_ENREF_100)] |
| Asia, East | [[12](#_ENREF_12),[100](#_ENREF_100)] |
| Asia, South | [[100](#_ENREF_100),[101](#_ENREF_101),[102](#_ENREF_102),[103](#_ENREF_103),[104](#_ENREF_104)] |
| Asia, Southeast | [[16](#_ENREF_16),[105](#_ENREF_105)] |
| Australasia | [[100](#_ENREF_100),[106](#_ENREF_106),[107](#_ENREF_107),[108](#_ENREF_108)] |
| Europe, Central | [[100](#_ENREF_100),[109](#_ENREF_109),[110](#_ENREF_110)] |
| Europe, Eastern | [[100](#_ENREF_100)] |
| Europe, Western | [[30](#_ENREF_30),[49](#_ENREF_49),[51](#_ENREF_51),[55](#_ENREF_55),[100](#_ENREF_100),[111](#_ENREF_111),[112](#_ENREF_112),[113](#_ENREF_113),[114](#_ENREF_114),[115](#_ENREF_115),[116](#_ENREF_116),[117](#_ENREF_117),[118](#_ENREF_118),[119](#_ENREF_119),[120](#_ENREF_120),[121](#_ENREF_121),[122](#_ENREF_122)] |
| Latin America, Central | [[100](#_ENREF_100),[123](#_ENREF_123)] |
| Latin America, Southern | [[124](#_ENREF_124)] |
| Latin America, Tropical | [[60](#_ENREF_60),[100](#_ENREF_100)] |
| North Africa/Middle East | [[65](#_ENREF_65),[100](#_ENREF_100),[123](#_ENREF_123),[125](#_ENREF_125),[126](#_ENREF_126),[127](#_ENREF_127),[128](#_ENREF_128)] |
| North America, High Income | [[77](#_ENREF_77),[99](#_ENREF_99),[100](#_ENREF_100),[123](#_ENREF_123),[129](#_ENREF_129),[130](#_ENREF_130),[131](#_ENREF_131),[132](#_ENREF_132)] |
| Sub-Saharan Africa, East | [[133](#_ENREF_133),[134](#_ENREF_134),[135](#_ENREF_135),[136](#_ENREF_136)] |
| Sub-Saharan Africa, Southern | [[100](#_ENREF_100),[136](#_ENREF_136),[137](#_ENREF_137)] |
| Sub-Saharan Africa, West | [[100](#_ENREF_100),[138](#_ENREF_138)] |

1. Low prevalence disorders

| **Region** | **References for prevalence data sources** |
| --- | --- |
| **Schizophrenia** | |
| Asia Pacific, High Income | [[139](#_ENREF_139),[140](#_ENREF_140),[141](#_ENREF_141),[142](#_ENREF_142)] |
| Asia, East | [[143](#_ENREF_143),[144](#_ENREF_144),[145](#_ENREF_145),[146](#_ENREF_146),[147](#_ENREF_147)] |
| Asia, South | [[148](#_ENREF_148),[149](#_ENREF_149),[150](#_ENREF_150)] |
| Asia, Southeast | [[151](#_ENREF_151)] |
| Australasia | [[108](#_ENREF_108),[152](#_ENREF_152)] |
| Caribbean | [[22](#_ENREF_22),[153](#_ENREF_153)] |
| Europe, Eastern | [[154](#_ENREF_154),[155](#_ENREF_155)] |
| Europe, Western | [[47](#_ENREF_47),[112](#_ENREF_112),[156](#_ENREF_156),[157](#_ENREF_157),[158](#_ENREF_158),[159](#_ENREF_159),[160](#_ENREF_160),[161](#_ENREF_161),[162](#_ENREF_162),[163](#_ENREF_163),[164](#_ENREF_164),[165](#_ENREF_165),[166](#_ENREF_166),[167](#_ENREF_167),[168](#_ENREF_168),[169](#_ENREF_169),[170](#_ENREF_170),[171](#_ENREF_171),[172](#_ENREF_172),[173](#_ENREF_173),[174](#_ENREF_174),[175](#_ENREF_175),[176](#_ENREF_176)] |
| Latin America, Central | [[177](#_ENREF_177)] |
| North America, High Income | [[178](#_ENREF_178),[179](#_ENREF_179),[180](#_ENREF_180)] |
| Oceania | [[181](#_ENREF_181)] |
| Sub-Saharan Africa, East | [[86](#_ENREF_86),[133](#_ENREF_133),[182](#_ENREF_182)] |
| Sub-Saharan Africa, Southern | [[183](#_ENREF_183)] |
| **Bipolar Disorders** | |
| Asia Pacific, High Income | [[1](#_ENREF_1),[3](#_ENREF_3),[4](#_ENREF_4)] |
| Asia, East | [[7](#_ENREF_7),[10](#_ENREF_10),[12](#_ENREF_12),[184](#_ENREF_184)] |
| Australasia | [[20](#_ENREF_20),[185](#_ENREF_185)] |
| Europe, Eastern | [[29](#_ENREF_29)] |
| Europe, Western | [[30](#_ENREF_30),[39](#_ENREF_39),[45](#_ENREF_45),[47](#_ENREF_47),[54](#_ENREF_54),[55](#_ENREF_55),[116](#_ENREF_116),[118](#_ENREF_118),[186](#_ENREF_186)] |
| Latin America, Central | [[98](#_ENREF_98),[187](#_ENREF_187)] |
| Latin America, Southern | [[188](#_ENREF_188)] |
| Latin America, Tropical | [[60](#_ENREF_60)] |
| North Africa/Middle East | [[63](#_ENREF_63),[66](#_ENREF_66),[189](#_ENREF_189)] |
| North America, High Income | [[74](#_ENREF_74),[77](#_ENREF_77),[190](#_ENREF_190),[191](#_ENREF_191),[192](#_ENREF_192)] |
| Sub-Saharan Africa, East | [[86](#_ENREF_86)] |
| Sub-Saharan Africa, West | [[95](#_ENREF_95)] |
| **Eating disorders** | |
| Asia Pacific, High Income | [[1](#_ENREF_1)] |
| Asia, East | [[10](#_ENREF_10),[193](#_ENREF_193)] |
| Australasia | [[100](#_ENREF_100)] |
| Europe, Central | [[100](#_ENREF_100),[194](#_ENREF_194),[195](#_ENREF_195)] |
| Europe, Western | [[30](#_ENREF_30),[55](#_ENREF_55),[100](#_ENREF_100),[112](#_ENREF_112),[113](#_ENREF_113),[116](#_ENREF_116),[122](#_ENREF_122),[196](#_ENREF_196),[197](#_ENREF_197),[198](#_ENREF_198),[199](#_ENREF_199),[200](#_ENREF_200),[201](#_ENREF_201),[202](#_ENREF_202),[203](#_ENREF_203),[204](#_ENREF_204),[205](#_ENREF_205),[206](#_ENREF_206),[207](#_ENREF_207)] |
| Latin America, Central | [[100](#_ENREF_100)] |
| North Africa/Middle East | [[208](#_ENREF_208)] |
| North America, High Income | [[100](#_ENREF_100),[209](#_ENREF_209),[210](#_ENREF_210)] |
| Sub-Saharan Africa, East | [[211](#_ENREF_211)] |

**References**

1. Cho MJ, Kim JK, Jeon HJ, Suh T, Chung IW, et al. (2007) Lifetime and 12-month prevalence of DSM-IV psychiatric disorders among Korean adults. J Nerv Ment Dis 195: 203-210.

2. Fones CS, Kua EH, Ng TP, Ko SM (1998) Studying the mental health of a nation: a preliminary report on a population survey in Singapore. Singapore Med J 39: 251–255.

3. Ihara K, Muraoka Y, Oiji A, Nadaoka T (1998) Prevalence of mood disorders according to DSM-III-R criteria in the community elderly residents in Japan. Environmental Health and Preventive Medicine 3: 44–49.

4. Kawakami N, Shimizu H, Haratani T, Iwata N, Kitamura T (2004) Lifetime and 6-month prevalence of DSM-III-R psychiatric disorders in an urban community in Japan. Psychiatry Res 121: 293-301.

5. Kawakami N, Takeshima T, Ono Y, Uda H, Hata Y, et al. (2005) Twelve-month prevalence, severity, and treatment of common mental disorders in communities in Japan: Preliminary finding from the World Mental Health Japan Survey 2002-2003. Psychiatry Clin Neurosci 59: 441-452.

6. Nakao M, Yano E (2006) Somatic symptoms for predicting depression: One-year follow-up study in annual health examinations. Psychiatry Clin Neurosci 60: 219–225.

7. Shen Y, Zhang M, Huang Y, He Y, Liu Z, et al. (2006) Twelve-month prevalence, severity and unmet need for treatment of mental disorders in metropolitan China. Psychol Med 36: 257-267.

8. Chen R, Hu Z, Qin X, Xu X, Copeland JRM (2004) A community-based study of depression in older people in Hefei, China – The GMS-AGECAT prevalence, case validation and socio-economic correlates. Int J Geriatr Psychiatry 19: 407–413.

9. Hwu H-G, Chang IH, Yeh E-K, Chang C-J, Yeh L-L (1996) Major depressive disorder in Taiwan defined by the Chinese Diagnostic Interview Schedule. J Nerv Ment Dis 184: 497–502.

10. Keqing L, Ze C, Lijun C, Qinpu J, Guang S, et al. (2008) Epidemiological survey of mental disorders in the people aged 18 and older in Hebei province. Asian Journal of Psychiatry 1: 51–55.

11. Lu J, Ruan Y, Huang Y, Yao J, Dang W, et al. (2008) Major depression in Kunming: Prevalence, correlates and co-morbidity in a south-western city of China. J Affect Disord 111: 221–226.

12. Phillips MR, Zhang J, Shi Q, Song Z, Ding Z, et al. (2009) Prevalence, treatment, and associated disability of mental disorders in four provinces in China during 2001–05: an epidemiological survey. The Lancet 373: 2041–2053.

13. Simon GE, Goldberg D, Von Korff M, Ustun T (2002) Understanding cross-national differences in depression prevalence. Psychol Med 32: 585–594.

14. Nisar N, Billoo N, Gadit AA (2004) Prevalence of depression and the associated risks factors among adult women in a fishing community. Journal of the Pakistan Medical Association 54: 519–525.

15. Subedi S, Tausig M, Subedi J, Broughton CL, Williams-Blangero S (2004) Mental Illness and Disability among Elders in Developing Countries: The Case of Nepal. J Aging Health 16: 71–87.

16. Nguyen TTN, Long TK, Bui NL, Vos T, Ngo DA, et al. (2011) Viet Nam Burden of Disease and Injury Study. Hanoi.

17. Australian Bureau of Statistics (2008) National Survey of Mental Health and Wellbeing: Summary of results; ABS, editor. Canberra: Australian Bureau of Statistics.

18. Feehan M, McGee R, Raja SN, Williams SM (1994) DSM-III-R disorders in New Zealand 18-year-olds. Aust N Z J Psychiatry 28: 87–99.

19. Hawthorne G, Goldney R, Taylor AW (2008) Depression prevalence: Is it really increasing? . The Australian and New Zealand Journal of Psychiatry 42: 606–616.

20. Wells JE, Oakley Browne MA, Scott KM, McGee MA, Baxter J, et al. (2006) Prevalence, interference with life and severity of 12 month DSM-IV disorders in Te Rau Hinengaro: The New Zealand Mental Health Survey. Aust N Z J Psychiatry 40: 845–854.

21. Wilhelm K, Mitchell P, Slade T, Brownhill S, Andrews G (2003) Prevalence and correlates of DSM-IV major depression in an Australian national survey. J Affect Disord 75: 155–162.

22. Canino GJ, Bird HR, Shrout PE, Rubio-Stipec M, Bravo M, et al. (1987) The prevalence of specific psychiatric disorders in Puerto Rico. Arch Gen Psychiatry 44: 727–735.

23. Maharaj RG (2008) Depression among adolescents, aged 13–19 years, attending secondary schools in Trinidad. Prevalence and associated factors. West Indian Med J 57: 352–359.

24. Andrade L, Caraveo-Anduaga JJ, Berglund P, Bijl RV, De Graaf R, et al. (2003) The epidemiology of major depressive episodes: Results from the International Consortium of Psychiatric Epidemiology (ICPE) Surveys. International Journal of Methods in Psychiatric Research 12: 3–21.

25. Basoglu M, Livanou M, Crnobaric C, Franciskovic T, Suljic E, et al. (2005) Psychiatric and cognitive effects of war in former Yugoslavia: Association of lack of redress for trauma and posttraumatic stress reactions. JAMA: The Journal of the American Medical Association 294: 580–590.

26. Szadoczky E, Papp Z, Vitrai J, Rihmer Z, Furedi J (1998) The prevalence of major depressive and bipolar disorders in Hungary. Results from a national epidemiologic survey. J Affect Disord 50: 153–162.

27. Aluoja A, Leinsalu M, Shlik J, Vasar V, Luuk K (2004) Symptoms of depression in the Estonian population: prevalence, sociodemographic correlates and social adjustment. J Affect Disord 78: 27–35.

28. Bromet EJ, Gluzman SF, Paniotto VI, Webb CPM, Tintle NL, et al. (2005) Epidemiology of psychiatric and alcohol disorders in Ukraine: Findings from the Ukraine World Mental Health Survey. Soc Psychiatry Psychiatr Epidemiol 40: 681-690.

29. Pakriev S, Vasar V, Aluoja A, Saarma M, Shlik J (1998) Prevalence of mood disorders in the rural population of Udmurtia. Acta Psychiatr Scand 97: 169–174.

30. Aalto-Setala T, Marttunen M, Tuulio-Henriksson A, Poikolainen K, Lonnqvist J (2001) One-month prevalence of depression and other DSM-IV disorders among young adults. Psychol Med 31: 791-801.

31. Angst J, Merikangas K, Scheidegger P, Wicki W (1990) Recurrent brief depression: a new subtype of affective disorder. J Affect Disord 19: 87–98.

32. Angst J, Wicki W (1991) The Zurich study. XI. Is dysthymia a separate form of depression? Results of the Zurich Cohort Study. Eur Arch Psychiatry Clin Neurosci 240: 349–354.

33. Ayuso-Mateos JL, Vazques-Barquero JL, Dowrick C, Lehtinen V, Dalgard OS, et al. (2001) Depressive disorders in Europe: Prevalence figures from the ODIN study. Br J Psychiatry 179: 308–316.

34. Barry MM, Van Lente E, Molcho M, Morgan K, McGee H, et al. (2009) SLAN 2007: Survey of lifestyle, attitudes and nutrition in Ireland. Mental Health and Social Well-being Report. Dublin: Department of Health and Children.

35. Beekman ATF, Deeg DJJ, Van Tilburg T, Smith JH, Hooijer C, et al. (1995) Major and minor depression in later life: A study of prevalence and risk factors. J Affect Disord 36: 65–75.

36. Bracke P (1998) Sex differences in the course of depression: evidence from a longitudinal study of a representative sample of the Belgian population. Soc Psychiatry Psychiatr Epidemiol 33: 420–429.

37. Carta MG, Kovess V, Hardoy MC, Morosini P, Murgia S, et al. (2002) Psychiatric disorders in Sardinian immigrants to Paris: A comparison with Parisians and Sardinians resident in Sardinia. Soc Psychiatry Psychiatr Epidemiol 37: 112–117.

38. Copeland JRM, Beekman ATF, Dewey ME, Hooijer C, Jordan A, et al. (1999) Depression in Europe. Geographical distribution among older people. Br J Psychiatry 174: 312–321.

39. Faravelli C, Degl'Innocenti BG, Aiazzi L, Incerpi G, Pallanti S (1990) Epidemiology of mood disorders: A community survey in Florence. J Affect Disord 20: 135–141.

40. Frojd S, Marttunen M, Pelkonen M, von der Pahlen B, Kaltiala-Heino R (2007) Adult and peer involvement in help-seeking for depression in adolescent population. A two-year follow-up in Finland. Soc Psychiatry Psychiatr Epidemiol 42: 945–952.

41. Godin O, Dufouil C, Ritchie K, Dartigues JF, Tzourio C, et al. (2007) Depressive symptoms, major depressive episode and cognition in the elderly: The three-city study. Neuroepidemiology 28: 101–108.

42. Jenkins R, Lewis G, Bebbinton P, Brugha T, Farrell M, et al. (1997) The National Psychiatric Morbidity Surveys of Great Britain – Initial findings from the Household Survey. . Psychol Med 27: 775–789.

43. Jylha P, Isometsa E (2006) The relationship of neuroticism and extraversion to symptoms of anxiety and depression in the general population. Depress Anxiety 23: 281–289.

44. Kirby M, Bruce I, Radic A, Coakley D, Lawlor BA (1997) Mental disorders among the community-dwelling elderly in Dublin. Br J Psychiatry 171: 369–372.

45. Kringlen E, Torgensen S, Cramer V (2001) A Norwegian psychiatric epidemiological study. AJ Psychiatry 158: 1091-1098.

46. Lepine J, Gastpar M, Mendlewicz J, Tylee A (1997) Depression in the community: The first pan-European study. DEPRES (Depression Research in European Society). Int Clin Psychopharmacol 12: 19–29.

47. Levav I, Kohn R, Dohrenwend BP, Shrout PE, Skodol AE, et al. (1993) An epidemiological study of mental disorders in a 10-year cohort of young adults in Israel. Psychol Med 23: 691–707.

48. Pahkala K, Kesti E, Kongas-Saviaro P, Laippala P, Kivela SL (1995) Prevalence of depression in an aged population in Finland. Soc Psychiatry Psychiatr Epidemiol 30: 99–106.

49. Pirkola SP, Isometsa E, Suvisaari J, Aro H, Joukamaa M, et al. (2005) DSM-IV mood-, anxiety- and alcohol use disorders and their comorbidity in the Finnish general population. Results from the Health 2000 Study. Soc Psychiatry Psychiatr Epidemiol 40: 1-10.

50. Ponizovsky AM, Grinshpoon A (2009) Mood and anxiety disorders and the use of services and psychotropic medication in an immigrant population: Findings from the Israel National Health Survey. Canadian Journal of Psychiatry 54: 2009/06/17:409–419.

51. Ritchie K, Artero S, Beluche I, Ancelin ML, Mann A, et al. (2004) Prevalence of DSM-IV psychiatric disorder in the French elderly population. Br J Psychiatry 184: 147–152.

52. Saunders PA, Copeland JRM, Dewey ME, Gilmore C, Larkin BA, et al. (1993) The prevalence of dementia, depression and neurosis in later life: The Liverpool MRC-ALPHA study. Int J Epidemiol 22: 838–847.

53. Singleton N, Bumpstead R, O'Brien M, Lee A, Meltzer H (2001) Psychiatric morbidity among adults living in private households. London: The Stationery Office.

54. Stefansson GE, Bjornsson JK, Gudmundsdottir A (1994) Period prevalence rates of specific mental disorders in an Icelandic cohort. Soc Psychiatry Psychiatr Epidemiol 29: 119–125.

55. Verhulst FC, Van der Ende J, Ferdinand RF, Kasius MC (1997) The prevalence of DSM-III-R diagnoses in a national sample of dutch adolescents. Arch Gen Psychiatry 54: 329–336.

56. Weissman MM, Bland RC, Canino GJ, Faravelli C, Greenwald S, et al. (1996) Cross-national epidemiology of major depression and bipolar disorder. JAMA: The Journal of the American Medical Association 276: 293–299.

57. Kohn R, Levav I, Garcia ID, Machuca ME, Tamashiro R (2005) Prevalence, risk factors and aging vulnerability for psychopathology following a natural disaster in a developing country. Int J Geriatr Psychiatry 20: 2005/08/24:835–841.

58. Slone LB, Norris FH, Murphy AD, Baker CK, Perilla JL, et al. (2006) Epidemiology of major depression in four cities in Mexico. Depress Anxiety 23: 158–167.

59. Araya R, Rojas G, Fritsch R, Acuna J, Lewis G (2001) Common mental disorders in Santiago, Chile: Prevalence and socio-demographic correlates. Br J Psychiatry 178: 228–233.

60. Andrade L, Walters E, Gentil V, Laurenti R (2002) Prevalence of ICD-10 mental disorders in a catchment area in the city of Sao Paulo, Brazil. Soc Psychiatry Psychiatr Epidemiol 3: 316–325.

61. Costa E, Barreto SM, Uchoa E, Firmo JOA, Lima-Costa MF, et al. (2007) Prevalence of International Classification of Diseases, 10th Revision common mental disorders in the elderly in a Brazilian community: The Bambui health ageing study. Am J Geriatr Psychiatry 15: 17–27.

62. Afifi M, Al Riyami A, Morsi M, Al Kharusil H, Afifi M, et al. (2006) Depressive symptoms among high school adolescents in Oman. Eastern Mediterranean Health Journal 12 Suppl 2: S126–S137.

63. Alhasnawi A, Sadik S, Rasheed M, Baban A, Al-Alak MM, et al. (2009) The prevalence and correlates of DSM-IV disorders in the Iraq mental health survey (IHMS). World Psychiatry 8: 97–109.

64. Bostanci M, Ozdel O, Oguzhanoglu NK, Ozdel L, Ergin A, et al. (2005) Depressive symptomatology among university students in Denizli, Turkey: prevalence and sociodemographic correlates. Croat Med J 46: 96–100.

65. Ghanem M, Gadallah M, Meky FA, Mourad S, El-Kholy G (2009) National survey of prevalence of mental disorders in Egypt: Preliminary survey. Eastern Mediterranean Health Journal 15: 65–75.

66. Karam EG, Mneimneh ZN, Karam AN, Fayyad JA, Nasser SC, et al. (2006) Prevalence and treatment of mental disorders in Lebanon: A national epidemiological survey. The Lancet 367: 1000–1006.

67. Lopes Cardozo B, Bilukha OO, Gotway CA, Wolfe MI, Gerber ML, et al. (2005) Mental health of women in postwar Afghanistan. Journal of Women's Health 14: 285-293.

68. Scholte WF, Olff M, Ventevogel P, De Vries GJ, Jansveld E, et al. (2004) Mental health symptoms following war and repression in Eastern Afghanistan. Journal of the American Medical Association 292: 585–593.

69. Bland R, Newman S, Orn H (1988) Period prevalence of psychiatric disorders in Edmonton. Acta Psychiatr Scand 77: 33–42.

70. Bland RC, Newman SC, Orn H (1988) Prevalence of psychiatric disorders in the elderly in Edmonton. Acta Psychiatr Scand 77: 57-63.

71. Bland RC, Newman SC, Orn H (1997) Age and remission of psychiatric disorders. Canadian Journal of Psychiatry 42: 722–729.

72. Blazer DG, Kessler RC, McGonagle KA, Swartz MS (1994) The prevalence and distribution of major depression in a national community sample: The National Comorbidity Survey. AJ Psychiatry 151: 979–986.

73. Cohen P, Cohen J, Kasen S, Velez CN, Hartmark C, et al. (1993) An epidemiological study of disorders in late childhood and adolescence – I. Age- and gender-specific prevalence. J Child Psychol Psychiatry 34: 851–867.

74. Gum AM, King-Kallimanis B, Kohn R (2009) Prevalence of mood, anxiety, and substance-abuse disorders for older Americans in the National Comorbidity Survey-replication. The American Journal of Geriatric Psychiatry 17: 769–781.

75. Kessler RC, McGonagle KA, Swartz M, Blazer DG, Nelson CB (1993) Sex and depression in the National Comorbidity Survey: I. Lifetime prevalence, chronicity and recurrence. J Affect Disord 29: 85–96.

76. Kessler RC, Walters EE (1998) Epidemiology of DSM-III-R major depression and minor depression among adolescents and young adults in the National Comorbidity Survey. Depress Anxiety 7: 3–14.

77. Lewinsohn PM, Hops H, Roberts RE, Seeley JR, Andrews JA (1993) Adolescent psychopathology: I. Prevalence and incidence of depression and other DSM-III-R disorders in high school students. J Abnorm Psychol 102: 133–144.

78. Mojtabai R, Olfson M (2004) Major depression in community-dwelling middle-aged and older adults: prevalence and 2- and 4-year follow-up symptoms. Psychol Med 34: 623–634.

79. Newman SC, Bland RC, Orn HT (1998) The prevalence of mental disorders in the elderly in Edmonton: a community survey using GMS-AGECAT. Geriatric Mental State-Automated Geriatric Examination for Computer Assisted Taxonomy. Canadian Journal of Psychiatry 43: 910–914.

80. Offord DR, Boyle MH, Campbell D, Goering P, Lin E, et al. (1996) One-year prevalence of psychiatric disorder in Ontarians 15 to 64 years of age. . Canadian Journal of Psychiatry 41: 559–563.

81. Patten SB (2001) The duration of major depressive episodes in the Canadian general population. Chronic Dis Can 22: 6–11.

82. Patten SB, Stuart HL, Russell ML, Maxwell CJ, Arboleda-Florez J (2003) Epidemiology of major depression in a predominantly rural health region. Soc Psychiatry Psychiatr Epidemiol 38: 360–365.

83. Regier DA, Boyd JH, Burke JD, Jr., Rae DS, Myers JK, et al. (1988) One-month prevalence of mental disorders in the United States. Based on five Epidemiologic Catchment Area sites. Arch Gen Psychiatry 45: 977–986.

84. Bolton P, Neugebauer R, Ndogoni L (2002) Prevalence of depression in rural Rwanda based on symptom and functional criteria. J Nerv Ment Dis 190: 631–637.

85. Bolton P, Wilk CM, Ndogoni L (2004) Assessment of depression prevalence in rural Uganda using symptom and function criteria. Soc Psychiatry Psychiatr Epidemiol 39: 442–447.

86. Kebede D, Alem A (1999) Major mental disorders in Addis Ababa, Ethiopia. II. Affective disorders. Acta Psychiatr Scand 100: 18–23.

87. Ovuga E, Boardman J, Wasserman D (2005) The prevalence of depression in two districts of Uganda. Soc Psychiatry Psychiatr Epidemiol 40: 439–445.

88. Roberts B, Damundu EY, Lomoro O, Sondorp E (2009) Post-conflict mental health needs: a cross-sectional survey of trauma, depression and associated factors in Juba, Southern Sudan. BMC Psychiatry 9: 7.

89. Shaaban KMA, Baashar TA (2003) A community study of depression in adolescent girls: Prevalence and its relation to age. Medical Principles and Practice 12: 256–259.

90. Bhagwanjee A, Parekh A, Paruk Z, Petersen I, Subedar H (1998) Prevalence of minor psychiatric disorders in an adult African rural community in South Africa. Psychol Med 28: 1137–1147.

91. Hollifield M, Katon W, Spain D, Pule L (1990) Anxiety and depression in a village in Lesotho, Africa: A comparison with the United States. Br J Psychiatry 156: 343–350.

92. Adewuya AO, Ola BA, Aloba OO, Mapayi BM, Oginni OO (2006) Depression amongst Nigerian university students. Prevalence and sociodemographic correlates. Soc Psychiatry Psychiatr Epidemiol 41: 674–678.

93. Amoran O, Lawoyin T, Lasebikan V (2007) Prevalence of depression among adults in Oyo State, Nigeria: A comparative study of rural and urban communities. Aust J Rural Health 15: 211–215.

94. Coleman R, Morison L, Paine K, Powell RA, Walraven G (2006) Women's reproductive health and depression: A community survey in the Gambia, West Africa. Soc Psychiatry Psychiatr Epidemiol 41: 720–727.

95. Gureje O, Lasebikan VO, Kola L, Makanjuola VA (2006) Lifetime and 12-month prevalence of mental disorders in the Nigerian Survey of Mental Health and Well-Being. Br J Psychiatry 188: 465–471.

96. Uwakwe R (2000) The pattern of psychiatric disorders among the aged in a selected community in Nigeria. Int J Geriatr Psychiatry 15: 355–362.

97. Andrews G, Henderson S, Hall W (2001) Prevalence, comorbidity, disability and service utilisation. Overview of the Australian National Mental Health Survey. The British Journal of Psychiatry 178: 145-153.

98. Medina-Mora ME, Borges G, Lara C, Benjet C, Blanco J, et al. (2005) Prevalence, service use, and demographic correlates of 12-month DSM-IV psychiatric disorders in Mexico: Results from the Mexican National Comorbidity Survey. Psychol Med 35: 1773–1783.

99. Kessler RC, McGonable KA, Zao S, Nelson CB, Hughes M, et al. (1994) Lifetime and 12-month prevalence of DSM-III-R psychiatric disorders in the United States; results from the National Comorbidity Survey. Archives of General Psychiatry 51: 8-19.

100. WMHS Consortium (2008) Unpublished data provided by the World Mental Health Survey (WMHS) Consortium, December 2008.

101. Islam MM, Ali M, Ferroni P, Underwood P, Alam MF (2003) Prevalence of psychiatric disorders in an urban community in Bangladesh. Gen Hosp Psychiatry 25: 353–357.

102. Joshi K, Kumar R, Avasthi A (2003) Morbidity profile and its relationship with disability and pyschological distress among elderly people in Northern India. Int J Epidemiol 32: 978–987.

103. Hosain MGM, Chatterjee N, Ara N, Islam T (2007) Prevalence, pattern and determinants of mental disorders in rural Bangladesh. Public Health 121: 18–24.

104. Premarajan KC, Danabalan M, Chandrasekar R, Srinivasa DK (1993) Prevalence of psychiatry morbidity in an urban community of Pondicherry. Indian Journal of Psychiatry 35: 99–102.

105. Krishnaswamy S, Kavitha S, Aziz JA, H. I, Y. LW, et al. (2009) Burden, determinant and impact of common mental disorders in Malaysia. Under review.

106. Australian Bureau of Statistics (1997) National Survey of Mental Health and Wellbeing, 1997 (SMHWB) In: Statistics ABo, editor. Australian Bureau Statistics. Canberra: Australian Bureau of Statistics. pp. 1–11.

107. McEvoy PM, Grove R, Slade T (2011) Epidemiology of anxiety disorders in the Australian general population: findings of the 2007 Australian National Survey of Mental Health and Wellbeing. Aust N Z J Psychiatry 45: 957-967.

108. Oakley-Browne MA, Joyce PR, Wells JE, Bushnell JA, Hornblow AR (1989) Christchurch psychiatric epidemiology study, II: six month and other period prevelances for specific psychiatric disorders. Aust N Z J Psychiatry 23: 327-340.

109. Eytan A, Gex-Fabry M, Toscani L, Deroo L, Loutan L, et al. (2004) Determinants of postconflict symptoms in Albanian Kosovars. J Nerv Ment Dis 192: 664–671.

110. Szadoczky E, Rozsa S, Zambori J, Furedi J (2004) Anxiety and mood disorders in primary care practice. International Journal of Psychiatry in Clinical Practice 8: 77–84.

111. Beekman AT, Bremmer MA, Deeg DJ, van Balkom AJ, Smit JH, et al. (1998) Anxiety disorders in later life: a report from the Longitudinal Aging Study Amsterdam. Int J Geriatr Psychiatry 13: 717–726.

112. Bijl RV, Ravelli A, van Zessen G (1998) Prevalence of psychiatric disorder in the general population: Results of the Netherlands Mental Health Survey and Incidence Study (NEMESIS). Soc Psychiatry Psychiatr Epidemiol 33: 587-595.

113. Canals J, Domenech E, Carbajo G, Blade J (1997) Prevalence of DSM-III-R and ICD-10 psychiatric disorders in a Spanish population of 18-year-olds. Acta Psychiatr Scand 96: 287–294.

114. Carta MG, Carpiniello B, Morosini PL, Rudas N (1991) Prevalence of mental disorders in Sardinia: A community study in an inland mining district. Psychol Med 21: 1061–1071.

115. Faravelli C, Guerrini Degl'Innocenti B, Giardinelli L (1989) Epidemiology of anxiety disorders in Florence. Acta Psychiatr Scand 79: 308–312.

116. Faravelli C, Abrardi L, Bartolozzi D, Cecchi C, Cosci F, et al. (2004) The Sesto Fiorentino study: Point and one-year prevalences of psychiatric disorders in an Italian community sample using clinical interviewers. Psychother Psychosom 73: 226–234.

117. Gigantesco A, Palumbo G, Mirabella F, Pettinelli M, Morosini P (2006) Prevalence of psychiatric disorders in an Italian town: Low prevalence confirmed with two different interviews. Psychother Psychosom 75: 170–176.

118. Jacobi F, Wittchen HU, Holting C, Hofler M, Pfister H, et al. (2004) Prevalence, co-morbidity and correlates of mental disorders in the general population: results from the German Health Interview and Examination Survey (GHS). Psychol Med 34: 597–611.

119. McConnell P, Bebbington P, McClelland R, Gillespie K, Houghton S (2002) Prevalence of psychiatric disorder and the need for psychiatric care in northern Ireland: Population study in the district of Derry. Source British Journal of Psychiatry 181: 214–219.

120. Meyer C, Rumpf HJ, Hapke U, John U (2001) Prevalence of DSM-IV psychiatric disorders including nicotine dependence in the general population: Results from the Northern German TACOS study. Neurology Psychiatry and Brain Research 9: 75–80.

121. Sandanger I, Nygard JF, Ingebrigtsen G, Sorensen T, Dalgard OS (1999) Prevalence, incidence and age at onset of psychiatric disorders in Norway. Soc Psychiatry Psychiatr Epidemiol 34: 570–579.

122. Wittchen HU, Nelson CB, Lachner G (1998) Prevalence of mental disorders and psychosocial impairments in adolescents and young adults. Psychol Med 28: 109-126.

123. Andrade L, Caraveo-Anduaga JJ, Berglund P, Bijl R, Kessler RC, et al. (2000) Cross-national comparisons of the prevalences and correlates of mental disorders. Bulletin of the World Health Organisation 78: 413–426.

124. Vicente B, Kohn R, Rioseco P, Saldivia S, Baker C, et al. (2004) Population prevalence of psychiatric disorders in Chile: 6-month and 1-month rates. The British Journal Of Psychiatry 184: 299–305.

125. Ghubash R, Hamdi E, Bebbington P (1992) The Dubai Community Psychiatric Survey: Prevalence and socio-demographic correlates. Soc Psychiatry Psychiatr Epidemiol 27: 53–61.

126. Kadri N, Agoub M, El Gnaoui S, Berrada S, Moussaoui D (2007) Prevalence of anxiety disorders: A population-based epidemiological study in metropolitan area of Casablanca, Morocco. Annals of General Psychiatry 6: (epub).

127. Ventevogel P, De Vries G, Scholte WF, Shinwari NR, Faiz H, et al. (2007) Properties of the Hopkins Symptom Checklist-25 (HSCL-25) and the Self-Reporting Questionnaire (SRQ-20) as screening instruments used in primary care in Afghanistan. Soc Psychiatry Psychiatr Epidemiol 42: 328–335.

128. Yasan A, Saka G, Ozkan M, Ertem M (2009) Trauma type, gender and risk of PTSD in a region within an area of conflict. . J Trauma Stress 22: 663–666.

129. Breton JJ, Bergeron L, Valla JP, Berthiaume C, Gaudet N, et al. (1999) Quebec Child Mental Health Survey: prevalence of DSM-III-R mental health disorders. Journal of Child Psychology & Psychiatry 40: 375–384.

130. Narrow WE, Rae DS, Robins LN, Regier DA (2002) Revised prevalence estimates of mental disorders in the United States: Using a clinical significance criterion to reconcile 2 surveys' estimates. Arch Gen Psychiatry 59: 115–123.

131. Nguyen CT, Fournier L, Bergeron L, Roberge P, Barrette G (2005) Correlates of depressive and anxiety disorders among young Canadians. Canadian Journal of Psychiatry 50: 620–628.

132. Regier DA, Narrow WE, Rae DS (1990) The epidemiology of anxiety disorders: The Epidemiologic Catchment Area (ECA) experience. J Psychiatr Res 24 Suppl 2: 3–14.

133. Awas M, Kebede D, Alem A (1999) Major mental disorders in Butajira, southern Ethiopia. Acta Psychiatr Scand Suppl 397: 56–64.

134. Orley J, Wing JK (1979) Psychiatric disorders in two African villages. Arch Gen Psychiatry 36: 513–520.

135. Pham PN, Weinstein HM, Longman T (2004) Trauma and PTSD symptoms in Rwanda: implications for attitudes toward justice and reconciliation. JAMA: The Journal of the American Medical Association 292: 602–612.

136. Seedat S, Nyamai C, Njenga F, Vythilingum B, Stein DJ (2004) Trauma exposure and post-traumatic stress symptoms in urban African schools. Survey in CapeTown and Nairobi. The British Journal Of Psychiatry: The Journal Of Mental Science 184: 169-175.

137. Abas MA, Broadhead JC (1997) Depression and anxiety among women in an urban setting in Zimbabwe. Psychol Med 27: 59–71.

138. Adewuya AO, Ola BA, Adewumi TA (2007) The 12-month prevalence of DSM-IV anxiety disorders among Nigerian secondary school adolescents aged 13-18 years. J Adolesc 30: 17 Sep 2007:1071–1076.

139. Fujita T (1991) Trends of psychiatric in and out patients. Nippon Koshu Eisei Zasshi 38: 233-245.

140. Ichinowatari N, Tatsunuma T, Makiya H (1987) Epidemiological study of old age mental disorders in the two rural areas of Japan. JpnJPsychiatry Neurol 41: 629-636.

141. Nakamura Y, Ojima T, Oki I, Tanihara S, Yanagawa H (1997) Estimation of the future numbers of patients with mental disorders in Japan based on the results of National Patient Surveys. J Epidemiol 7: 214-220.

142. Suzuki M, Morita H, Kamoshita S (1990) Epidemiological survey of psychiatric disorders in Japanese school children. Part III: Prevalence of psychiatric disorders in junior high school children. Nippon Koshu Eisei Zasshi 37: 991-1000.

143. Chen C, Zhang WX (1998) Epidemiological survey on schizophrenia in 7 areas of China. Chin J Psychiatry 31: 72-74.

144. Phillips MR, Yang G, Li S, Li Y (2004) Suicide and the unique prevalence pattern of schizophrenia in mainland China: A retrospective observational study. The Lancet 364: 1062-1068.

145. Ran M, Xiang M, Li SX, Shan Y, Huang M, et al. (2003) Prevalence and course of schizophrenia in a Chinese rural area. Aust N Z J Psychiatry 37: 452-457.

146. Xu WS (1991) Analysis of the cultural and mental diseases rates of the three nationalities from She, Hui and Mongolian peoples. Chung Hua ShenChingChingShenKo Tsa Chih 24: 87-89, 124.

147. Hwu HG, Yeh EK, Chang LY (1989) Prevelance of psychiatric disorders in Taiwan defined by the Chinese diagnostic interview schedule. Acta Psychiatry Scandinavica 79: 136-147.

148. Mehta P, Joseph A, Verghese A (1985) An epidemiologic study of psychiatric disorders in a rural area in Tamilnadu. Indian Journal of Psychiatry 27: 153-158.

149. Padmavathi R, Rajkumar S, Kumar N, Manoharan A, Kamath S (1987) Prevalence of schizophrenia in an urban community in Madras. Indian Journal of Psychiatry 31: 233-239.

150. Sachdeva J, Singh S, Sidhu BS, Goyal RKD, Sing J (1986) An epidemiological study of psychiatric disorders in rural Faridkot. Indian Journal of Psychiatry 28: 317-323.

151. Salan R (1992) Epidemiology of schizophrenia in Indonesia (the Tambora I study). ASEAN Journal of Psychiatry 2: 52-57.

152. Jablensky A, McGrath J, Herrman H, Castle D, Gureje O, et al. (2000) Psychotic disorders in urban areas: an overview of the Study on Low Prevalence Disorders. . Aust N Z J Psychiatry 34: 221-236.

153. Kay RW (1989) Prevalence of psychotic mental disorders in the Commonwealth of Dominica. Abstract.

154. Shmaonova LM (1983) Possibilities of an Epidemiological Method and Some Results of a Population Study of Schizophrenia. Zh Nevropatol Psikhiatr Im S S Korsakova 83: 707-716.

155. Yursinova MS (1982) Some results of an epidemiological study of schizophrenia in the city of Samarkand. Zhurnal Nevropatologii i Psikhiatrii imeni SSKorsakova 93-98.

156. Bojholm S, Stromgren E (1989) Prevalence of schizophrenia on the island of Bornholm in 1935 and in 1983. Acta Psychiatr Scand Suppl 348: 157-166; discussion 167-178.

157. Jorda-Moscardo E, Munk-Jorgensen P (1986) A comparative census study of Danish schizophrenic patients in 1977 and 1982. Eur Arch Psychiatry Neurol Sci 235: 323-327.

158. Munk-Jorgensen P, Weeke A, Jensen EB, Dupont A, Stromgren E (1986) Changes in utilization of Danish psychiatric institutions. II. Census studies 1977 and 1982. Compr Psychiatry 27: 416-429.

159. Thomsen PH (1996) Schizophrenia with childhood and adolescent onset--a nationwide register-based study. Acta Psychiatr Scand 94: 187-193.

160. Cooper B, Sosna U (1983) Psychische Erkrankung in der Altenbevoelkerung. / Psychiatric illness in the elderly population. Nervenarzt 54: 239-249.

161. Keatinge C (1987) Schizophrenia in rural Ireland: a case of service overutilisation. Int J Soc Psychiatry 33: 186-194.

162. Youssef HA, Kinsella A, Waddington JL (1991) Evidence for geographical variations in the prevalence of schizophrenia in rural Ireland. Arch Gen Psychiatry 48: 254.

163. de Salvia D, Barbato A, Salvo P, Zadro F (1993) Prevalence and incidence of schizophrenic disorders in Portogruaro. An Italian case register study. Journal of Nervous and Mental Disorders 181: 275-282.

164. Hodiamont PP, Nelly; Syben, No (1987) Epidemiological aspects of psychiatric disorder in a Dutch health area. Psychol Med 17: 495-505.

165. Schrier AC, van de Wetering BJ, Mulder PG, Selten JP (2001) Point prevalence of schizophrenia in immigrant groups in Rotterdam: data from outpatient facilities. Eur Psychiatry 16: 162-166.

166. Grawe RW, Pedersen PB, Widen JH (1997) Changes in prevalence and comorbidity in a total population of patients with psychotic disorders in Norwegian psychiatric hospitals. Nord J Psychiatry 51: 127-132.

167. Jay M, Gorwood P, Feingold J, Leboyer M (1997) A one year prevalence study of schizophrenia on Reunion Island. Eur Psychiatry 12: 284-288.

168. Moreno-Kustner B, Rosales-Varo C, Torres-Gonzalez F, Emmett C (2002) The treated prevalence of schizophrenia in Granada. Data from the accummulative case register. Acta Psychiatr Scand 105: 24.

169. Lindstrom E, Widerlov B, von Knorring L (1997) The ICD-10 and DSM-IV diagnostic criteria and the prevalence of schizophrenia. Eur Psychiatry 12: 217-223.

170. Widerlov B, Borga P, Cullberg J, Stefansson CG, Lindqvist G (1989) Epidemiology of long-term functional psychosis in three different areas in Stockholm County. Acta Psychiatr Scand 80: 40-46.

171. Bamrah JS, Freeman HL, Goldberg DP (1991) Epidemiology of schizophrenia in Salford, 1974-84. Changes in an urban community over ten years. Br J Psychiatry 159: 802-810.

172. Goldacre M, Shiwach R, Yeates D (1994) Estimating incidence and prevalence of treated psychiatric disorders from routine statistics: the example of schizophrenia in Oxfordshire. J Epidemiol Community Health 48: 318-322.

173. Harvey CA, Pantelis C, Taylor J, McCabe PJ, Lefevre K, et al. (1996) The Camden schizophrenia surveys. II. High prevalence of schizophrenia in an inner London borough and its relationship to socio-demographic factors. Br J Psychiatry 168: 418-426.

174. Jeffreys SE, Harvey CA, McNaught AS, Quayle AS, King MB, et al. (1997) The Hampstead Schizophrenia Survey 1991. I: Prevalence and service use comparisons in an inner London health authority, 1986-1991. Br J Psychiatry 170: 301-306.

175. McCreadie RG, Leese M, Tilak-Singh D, Loftus L, MacEwan T, et al. (1997) Nithsdale, Nunhead and Norwood: similarities and differences in prevalence of schizophrenia and utilisation of services in rural and urban areas. Br J Psychiatry 170: 31-36.

176. Pantelis C, Taylor J, Campbell PG (1988) The South Camden schizophrenia survey - an experience of community-based research. Bulletin of the Royal College of Psychiatrists 12: 98-101.

177. Diaz-Martinez A, Diaz-Martinez R, Osornio-Rojo A, Rascon-Gasca ML (2003) Mental health in a Queretaro State, Mexico, municipality: A community psychiatric investigation model. Gac Med Mex 139: 101-107.

178. Kramer M, German PS, Anthony JC, Von Korff M, Skinner EA (1985) Patterns of mental disorders among the elderly residents of eastern Baltimore. J Am Geriatr Soc 33: 236-245.

179. Leaf PJ, Myers JK, McEvoy LT (1991) Procedures used in the epidemiologic catchment area study. In: Robins LN, Regier DA, editors. Psychiatric Disorders In America. New York: The Free Press.

180. Wu EQ, Shi L, Birnbaum H, Hudson T, Kessler R (2006) Annual prevalence of diagnosed schizophrenia in the USA: A claims data analysis approach. Psychol Med 36: 1535-1540.

181. Waldo MC (1999) Schizophrenia in Kosrae, Micronesia: prevalence, gender ratios, and clinical symptomatology. Schizophr Res 35: 175-181.

182. Bondestam S, Garssen J, Abdulwakil AI (1990) Prevalence and treatment of mental disorders and epilepsy in Zanzibar. Acta Psychiatr Scand 81: 327-331.

183. Ben-Tovim DI, Cushnie JM (1986) The prevalence of schizophrenia in a remote area of Botswana. Br J Psychiatry 148: 576-580.

184. Lee S, Ng KL, Tsang A (2009) A community survey of the twelve-month prevalence and correlates of bipolar spectrum disorder in Hong Kong. J Affect Disord 117: 79–86.

185. Mitchell PB, Slade T, Andrews G (2004) Twelve-month prevalence and disability of DSM-IV bipolar disorder in an Australian general population survey. Psychol Med 34: 777–785.

186. Scully PJ, Owens JM, Kinsella A, Waddington JL (2004) Schizophrenia, schizoaffective and bipolar disorder within an epidemiologically complete, homogeneous population in rural Ireland: Small area variation in rate. Schizophr Res 67: 143–155.

187. Benjet C, Borges G, Medina-Mora ME, Zambrano J, Aguilar-Gaxiola S (2009) Youth mental health in a populous city of the developing worlds: results from the Mexican Adolescent Mental health Survey. . The Journal of Child Psychology and Psychiatry 50: 386-395.

188. Vicente B, Rioseco P, Saldivia S, Kohn R, Torres S (2002) Chilean study on the prevalence of psychiatric disorders (DSM-III-R/CIDI) (ECPP). Rev Med Chil 130: 527-536.

189. Kadri N, Agoub M, Assouab F, Tazi MA, Didouh A, et al. (2010) Moroccan national study on prevalence of mental disorders: a community based epidemiological study. Acta Psychiatr Scand 121: 71–74.

190. Costello EJ (1989) Child psychiatric disorders and their correlates: A primary care pediatric sample. J Am Acad Child Adolesc Psychiatry 28: 851–856.

191. Lewinsohn PM, Klein DN, Seeley JR (1995) Bipolar disorders in a community sample of older adolescents: Prevalence, phenomenology, comorbidity, and course. J Am Acad Child Adolesc Psychiatry 34: 454–463.

192. Weissman MM, Leaf PJ, Tischler GL, Blazer DG, et al. (1988) Affective disorders in five United States communities. Psychol Med 18: 141–153.

193. Huon GF, Mingyi Q, Oliver K, Xiao G (2002) A large-scale survey of eating disorder symptomatology among female adolescents in the people's Republic of China. International Journal of Eating Disorders 32: 192-205.

194. Szumska I, Tury F, Csoboth CT, Rethelyi J, Purebl G, et al. (2005) The Prevalence of Eating Disorders and Weight-Control Methods among Young Women: A Hungarian Representative Study. European Eating Disorders Review 13: 278-284.

195. Tolgyes T, Nemessury J (2004) Epidemiological studies on adverse dieting behaviours and eating disorders among young people in Hungary. Soc Psychiatry Psychiatr Epidemiol 39: 647-654.

196. Mangweth-Matzek B, Rupp CI, Hausmann A, Assmayr K, Mariacher E, et al. (2006) Never too old for eating disorders or body dissatisfaction: a community study of elderly women. The International Journal of Eating Disorders 39: 583-586.

197. Fichter MM, Quadflieg N, Georgopoulou E, Xepapadakos F, Fthenakis EW (2005) Time Trends in Eating Disturbances in Young Greek Migrants. International Journal of Eating Disorders 38: 310-322.

198. Cotrufo P, Barretta V, Monteleone P, Maj M (1998) Full-syndrome, partial-syndrome and subclinical eating disorders: an epidemiological study of female students in Southern Italy. Acta Psychiatrica Scandinavica 98: 112-115.

199. Favaro A, Ferrara S, Santonastaso P (2003) The spectrum of eating disorders in young women: A prevalence study in a general population sample. Psychosomatic Medicine 65: 701-708.

200. Gotestam KG, Agras WS (1995) General population-based epidemiological study of eating disorders in Norway. International Journal of Eating Disorders 18: 119-126.

201. Heiervang E, Stormark KM, Lundervold AJ, Heimann M, Goodman R, et al. (2007) Psychiatric Disorders in Norwegian 8- to 10-Year-Olds: An Epidemiological Survey of Prevalence, Risk Factors, and Service Use. [Article]. J Am Acad Child Adolesc Psychiatry 46: 438-447.

202. Do Carmo I, Reis D, Varandas P, Bouca D, Padre Santo D, et al. (1996) Prevalence of anorexia nervosa: A Portuguese population study. European Eating Disorders Review 4: 157-170.

203. Machado PPP, Machado BC, GonÃ§alves Sn, Hoek HW (2007) The prevalence of Eating Disorders Not Otherwise Specified. Int J Eat Disord 40: 212-217.

204. Beato-Fernandez L, Rodriguez-Cano T, Belmonte-Llario A, Martinez-Delgado C (2004) Risk factors for eating disorders in adolescents: A Spanish community-based longitudinal study. European Child & Adolescent Psychiatry 13: 287-294.

205. Pelaez Fernandez MA, Labrador FJ, Raich RM (2007) Prevalence of eating disorders among adolescent and young adult scholastic population in the region of Madrid (Spain). Journal of Psychosomatic Research 62: 681-690.

206. Gual P, Perez-Gaspar M, Martinez-Gonzalez MA, Lahortiga F, de Irala-Estevez J, et al. (2002) Self-esteem, personality, and eating disorders: Baseline assessment of a prospective population-based cohort. International Journal of Eating Disorders 31: 261-273.

207. Ruiz-Lazaro PM, Alonso JP, Comet P, Lobo A, Velilla M (2005) Prevalence of Eating Disorders in Spain: A Survey on a Representative Sample of Adolescents. In: Swain PI, editor. Trends in eating disorders research: Hauppauge, NY, US: Nova Biomedical Books. . pp. 85-108.

208. Eapen V, Mabrouk AA, Bin-Othman S (2006) Disordered eating attitudes and symptomatology among adolescent girls in the United Arab Emirates. Eating Behaviors 7: 53-60.

209. Hudson JI, Hiripi E, Pope HG, Kessler RC (2007) The prevalence and correlates of eating disorders in the National Comorbidity Survey Replication. Biol Psychiatry 61: 348-358.

210. Lewinsohn PM, Striegel-Moore RH, Seeley JR (2000) Epidemiology and natural course of eating disorders in young women from adolescence to young adulthood. J Am Acad Child Adolesc Psychiatry 39: 1284-1292.

211. Eddy KT, Hennessey M, Thompson-Brenner H (2007) Eating pathology in East African women: The role of media exposure and globalization. Journal of Nervous and Mental Disease 195: 196-202.
