# Supplementary material for: Global Epidemiology of Mental Disorders: What Are We Missing?
Source: PLoS One. 2013 Jun 24;8(6):e65514. doi: 10.1371/journal.pone.0065514 (PMC3691161; doi:10.1371/journal.pone.0065514)
Supplement: Appendix S1 — Epidemiological measures required as data input. (DOCX) [file pone.0065514.s004.docx]

# Appendix S1: Epidemiological measures required as data input

**Prevalence**

Required information:

1. Prevalence of cases meeting DSM or ICD diagnostic criteria consistent with disorder definitions for GBD 2010. Point prevalence was defined as: Number of cases / Number of people in the population. Both the cases and the population are measured at the same point in time, and the cases are included in the population number.

Period prevalence was defined as: Number of people with disease during a certain period / Total Number of people in the population.

Estimates for prevalence were included if measures were reported as: point or 30-day prevalence, 3-month, 6-month or past-year prevalence.

2. Estimates of uncertainty (standard error (SE) or confidence interval (CI)) around prevalence. If SE or CI were not reported, SE was calculated using SE = (p*(1–p))/N where p was the proportion of cases and N the denominator.

**Incidence**

Required information:

1. Measures of the incidence hazard or the population rate (incidence probability) over a period of 12 months were required (i.e. estimates of annual incidence). The incidence hazard is defined as: Number of new cases with disease during a certain period / Total number of person time at risk. Persons are at risk when alive, susceptible, and not a case.

The population rate was defined as: Number of new cases with disease during a certain period / Average number of persons in the population. The “average number of persons in the population” serves as an approximation for the person-time at risk.

2. Estimates of uncertainty (standard error (SE) or confidence interval (CI)) around incidence.

**Remission**

Required information:

1. Remission was defined as *no longer fulfilling the diagnostic criteria for the disorder.* Remission is similarly defined as an annual hazard rate to recover from the disease, with person-time with disease in the denominator. These data are rarely available therefore where number of remitted cases were reported, the remission rate could be calculated: Weighted remission rate (%) = ∑ [a*((-ln (1 - b))/c)]/∑a where a is sample size, b is remission proportion and c is follow-up (years) where sufficient data were reported.

**Mortality**

Required information:

1. Measures of excess all-cause mortality were required in the form of either relative risk (RR), standardized mortality ratio (SMR) or hazard rate (HR). RR was defined as Mortality of diseased / Mortality of non-diseased. As mortality in mental disorders is relatively low, the denominator could be approximated by mortality of the total population.
