# Supplementary material for: Global Epidemiology of Mental Disorders: What Are We Missing?
Source: PLoS One. 2013 Jun 24;8(6):e65514. doi: 10.1371/journal.pone.0065514 (PMC3691161; doi:10.1371/journal.pone.0065514)
Supplement: Appendix S2 — Systematic search protocol. (DOCX) [file pone.0065514.s005.docx]

# Appendix S2: Systematic search protocol

Systematic reviews were conducted in 3 stages: searches of the peer-reviewed literature; searches for supplementary data described in key documents, online sources and the grey literature; and requests to mental health experts for additional material.

**4.1 Summary of inclusion criteria for studies :**

**For all parameters, inclusion criteria required that:**

- Sampling frames were representative of the community, such that it could reasonably be assumed that estimates reflect the population;
- ‘Caseness’ was based on internationally recognized diagnostic criteria (DSM or ICD);
- Disorders were defined as per categories required for GBD estimates, in the case of depression for example, separate estimates were required for MDD and for dysthymia while estimates for anxiety disorder must be for ‘any’ anxiety disorder;
- Data were obtained through primary data sources (i.e. not secondary sources such as a review article);
- Sufficient information were provided on study method and sample characteristics;
- No limitations on language of publication.

**Prevalence and incidence studies –**

- Prevalence data must be based on estimates for current (point or past month) or past-year prevalence: estimates of lifetime prevalence were not included;
- Incidence data must reflect annual incidence rates or at least provide sufficient data (i.e. person-years) so that annual incidence could be calculated;
- Incidence studies were prospective cohort or longitudinal studies.

**Remission and mortality studies -**

- Longitudinal follow-up must be naturalistic (ie. no specific intervention during follow-up period);
- Remission is defined as *no longer fulfilling the diagnostic criteria for the disorder*;
- The follow-up period must be at least 2 years;
- Case studies and studies that involved specific treatment trials were excluded;
- Mortality estimates reflected all-cause mortality;
- Studies were observational and analytic: case studies or treatment trials were excluded;
- Psychological autopsy studies were excluded.

**4.2 Peer-reviewed literature database search**

A broad search string was developed with the assistance of a research librarian to search relevant electronic databases (Medline, PsycINFO and EMBASE) and included both the broad term, for example in the case of anxiety disorders: ‘anxiety disorders’ and specific anxiety disorders AND epidemiolog* OR prevalen*. Searches were limited to ‘human’ studies published between 1980 and 2008. No limitations were set on language of publication. The broad search criteria elicited a huge number of articles, the title of each were reviewed and those that were of potential value were downloaded into an Endnote database for further review.

Abstracts of articles were scanned to identify papers containing epidemiological data and to identify duplicate records. Copies of the full text were obtained for remaining articles and these were read in greater detail to determine suitability for inclusion. Non-english language sources were translated with the assistance of staff and contacts at the University of Queensland and the IHME in Seattle. Where only broad age or person estimates were reported, authors were contacted to request more detailed estimates for males and females, preferably by age. Authors were also contacted for further information where required on study methodology such as years of data collection, sampling frames, parameter specifics (eg. prevalence period) and separate estimates for specific disorders such as MDD and dysthymia. Members of the research group met on a weekly basis to discuss issues with methodology and any uncertainty over interpretation of study findings.

***Example of search string used***

| **Electronic database search strings for eating eisorders** | |
| --- | --- |
| Incidence and Prevalence | |
| Medline and PsychINFO | (anorexia or bulimia or eating disorder*) and (prevalence or incidence) not animal* |
| EMBASE | (anorexia or bulimia or “eating disorder” or “eating disorders”) and (prevalence or incidence) |
| Remission and Mortality | |
| Medline and PsychINFO | (anorexia or bulimia or (eating disorder) or (eating disorders)) and (remission or relapse or duration or recovery or (life expectancy) or (cohort study) or (population study) or deaths or mortality or survival or (disease course)) |
| EMBASE | (anorexia or bulimia or ‘eating disorder’ or ‘eating disorders’) and (remission or relapse or ‘disease duration’ or recovery or ‘life expectancy’ or ‘cohort study’ or ‘population study’ or deaths or mortality or survival or ‘disease course’) |

**4.3 Search for additional data sources**

Additional searches were conducted of texts and key documents and online sources to identify further studies. Key websites were reviewed to identify any other data that may not have been published, such as national mental health surveys conducted by government statistical departments. Alternative academic databases, eg those with a focus on research from Africa or Asia, were searched for articles that may have been published in non-indexed journals and hence were not identified in the primary database search.

Alternative databases that were searched included :

- South African National Health Research Database (<http://www.researchdatabase.org.za/>)
- West African Research Association (<http://www.bu.edu/wara/>)
- The China Knowledge Resource Integrated Database

(<http://cnki.en.eastview.com/kns50/single_index.aspx>)

- Panteleimon (<http://www.panteleimon.org>)
- Google scholar (<http://google.com/scholar>)

Key documents such as the Mental Health Atlas produced by the World Health Organization [1], textbooks and previous review articles were identified and reference lists of these sources reviewed for additional studies not already identified.

**4.4 Expert consultation and requests to key experts and organisations**

Experts in the field were invited to review our list of studies identified to this point and asked to provide details of additional sources, including that available after 2008. Several large-scale collaborative groups have conducted cross-national surveys for mental disorders, in particular the International Consortium in Psychiatric Epidemiology (ICPE) and the more recent World Mental Health Survey Collaboration (WMHS). These groups were approached and invited to contribute data for member countries. No additional data were able to be provided for the ICPE studies, but several articles had been published on the studies conducted between 1990 and 1996 that allowed us to extract prevalence data for seven countries.

The WMHS Consortium was approached for results from their ongoing cross-national population surveys. The WMHS comprises national or regional household surveys in which structured surveys were administered between 2000 and 2009. Data were provided for age- and sex-specific prevalence of anxiety disorders for 22 countries [2].

References

1. World Health Organization (2005) Mental Health Atlas: 2005; WHO, Geneva.

2. World Mental health Survey Collaboration (2008) World Mental Health Survey, unpublished data. harvard University, Boston.

3. Cho MJ, Kim JK, Jeon HJ, Suh T, Chung IW, et al. (2007) Lifetime and 12-month prevalence of DSM-IV psychiatric disorders among Korean adults. Journal of Nervous and Mental Disease 195: 203–210.
