# Supplementary material for: Global Epidemiology of Mental Disorders: What Are We Missing?
Source: PLoS One. 2013 Jun 24;8(6):e65514. doi: 10.1371/journal.pone.0065514 (PMC3691161; doi:10.1371/journal.pone.0065514)
Supplement: Appendix S3 — Calculating population coverage. (DOCX) [file pone.0065514.s006.docx]

# Appendix S3: Calculating population coverage

Estimates of regional coverage for data were calculated by aggregating the available prevalence data for those aged 18-80 years, by disorder, country and GBD region. As our inclusion criteria required that study samples must be able to be generalizable to the population, we assumed that study age groups were representative of that age group within the largest geographical area represented in the country.

For example, where a country had two studies, with age groups ranging between 5-24 years and 16-65 years, the population represented within the data for that country were those aged 18-65 years. Therefore the first step was to calculate the proportion of the national population that was represented in the data based on the age groups covered in studies for that disorder. If studies for that country included only males or females, the national coverage was adjusted by the limited gender composition. Next, the national coverage was adjusted for geographical limitations of the data. For example, where a national study was conducted, the data were considered to be representative of the national population within the specific age groups covered by the data. If no national study was found, but several were conducted in various regions or provinces, the country coverage were adjusted by the proportional population for those provinces.

Once estimates for national coverage were arrived at, these were weighted by the population of the country relative to the population of the GBD region and summed to arrive at an estimate for regional coverage. All estimates were calculated with Microsoft Excel.

Example 1. National studies:

In High Income Asia Pacific there were 2 prevalence studies for anxiety disorders :

- A national Japan study for men and women aged 20 years of age and more [2]
- A national study in South Korea for persons aged 18 to 64 years [3]

As both studies reported national sampling frames, this data was considered to represent the national population for persons aged 20-80 years in Japan (91% of adult 18-80 year-old population) and 18-64 years in South Korea (89% of the 18-80 year-old population). The proportion of the population coverage for Japan and South Korea were weighted by the population of those countries relative to the population for Asia Pacific High Income (72% and 26%, respectively), resulting in a regional coverage for anxiety disorders, adjusted by study age groups and sampling frames, of 93%.

Example 2. Sub-national studies:

One study for anorexia was found for North Africa/Middle East, focussing on females aged 13-30 years from Al Ain Province province in the United Arab Emirates (UAE). As we decided to focus our calculations on the 18-80 year age group to improve comparability of estimates, we considered the target population to be females aged 18-30 years in the province of Al Ain.

In 2005 the proportion of the female UAE population aged 18-30 years was approximately 13% of the national population aged 18-80 years. As the sampling frame represented a single province, coverage was weighted by the proportion of national population within that province (8.5%), resulting in 1% estimated coverage of prevalence data for eating disorders in the adult population of UAE. As the UAE accounts for 1% of the population within North Africa/Middle East, we arrive at a regional estimate for coverage of anorexia of approximately one in 10,000 people, abbreviated to <0.1%.

Assumptions:

- Age groups, as a proportion of whole country populations, did not change between 1990 and 2005.

*Assumption tested using Chi-square analyses (available upon request).*

- The population within the study age group relative to the national population did not differ significantly between the period of data collection and reference year 2005.

*Assumption tested using Chi-square analyses (available upon request).*

- Where multiple studies were reported for a country, the most comprehensive study took priority, for example a national study was considered over sub-national studies.

#

References

1. World Health Organization (2005) Mental Health Atlas: 2005; WHO, Geneva.

2. World Mental health Survey Collaboration (2008) World Mental Health Survey, unpublished data. harvard University, Boston.

3. Cho MJ, Kim JK, Jeon HJ, Suh T, Chung IW, et al. (2007) Lifetime and 12-month prevalence of DSM-IV psychiatric disorders among Korean adults. Journal of Nervous and Mental Disease 195: 203–210.
